# Plantar soft tissues and Achilles tendon thickness and stiffness in people with diabetes: a systematic review

**DOI:** 10.1186/s13047-021-00475-7

**Published:** 2021-04-28

**Authors:** Benedictine Yen Chen Khor, James Woodburn, Lisa Newcombe, Ruth Barn

**Affiliations:** 1 Department of Podiatry and Radiography, School of Health and Life Sciences, Glasgow Caledonian University, Glasgow, G4 0BA Scotland, UK; 2 School of Health Sciences and Social Work, Griffith University, Queensland, Australia

**Keywords:** Achilles tendon, Diabetic foot, Diabetic foot ulcer, Diabetes related foot ulcer, Plantar soft tissues, Soft tissue properties, Tissue stiffness, Tissue thickness

## Abstract

**Background:**

Diabetes mellitus is associated with changes in soft tissue structure and function. However, the directionality of this change and the extent to which either tissue thickness or stiffness contributes to the pathogenesis of diabetes-related foot ulcerations is unclear. Hence, this systematic review aims to summarise the existing evidence for soft tissue structural differences in the feet of people with and without diabetes.

**Methods:**

In compliance with MOOSE and PRISMA guidelines, AMED, CINAHL, MEDLINE, ProQuest Health & Medical Collection, ProQuest Nursing & Allied Health Database, and Web of Science electronic databases were systematically searched for studies published from database inception until 1st October 2020 [Prospero CRD42020166614]. Reference lists of included studies were further screened. Methodological quality was appraised using a modified critical appraisal tool for quantitative studies developed by McMaster University.

**Results:**

A total of 35 non-randomised observational studies were suitable for inclusion. Within these, 20 studies evaluated plantar tissue thickness, 19 studies evaluated plantar tissue stiffness, 9 studies evaluated Achilles tendon thickness and 5 studies evaluated Achilles tendon stiffness outcomes. No significant differences in plantar tissue thickness were found between people with and without diabetes in 55% of studies (11/20), while significantly increased plantar tissue stiffness was found in people with diabetes in 47% of studies (9/19). Significantly increased Achilles tendon thickness was found in people with diabetes in 44% of studies (4/9), while no significant differences in Achilles tendon stiffness were found between people with and without diabetes in 60% of studies (3/5).

**Conclusions:**

This systematic review found some evidence of soft tissue structural differences between people with and without diabetes. However, uncertainty remains whether these differences independently contribute to diabetes-related foot ulcerations. The heterogeneity of methodological approaches made it difficult to compare across studies and methodological quality was generally inadequate. High-quality studies using standardised and validated assessment techniques in well-defined populations are required to determine more fully the role of structural tissue properties in the pathogenesis of diabetes-related foot ulcerations.

**Supplementary Information:**

The online version contains supplementary material available at 10.1186/s13047-021-00475-7.

## Introduction

Diabetes-related foot ulcerations (DFUs) are one of the most challenging complications of diabetes mellitus. They are associated with high morbidity and mortality [1, 2], with non-healing DFUs preceding up to 85% of all non-traumatic lower limb amputations [3], and are costly to healthcare systems [4]. Factors associated with the pathogenesis of DFUs are complex and multifactorial, but fundamentally involve the interaction of extrinsic biomechanical forces with intrinsic structural and functional properties of the skin and underlying soft tissues [5, 6].

Histological changes have been observed in the plantar soft tissues [7, 8] and Achilles tendon (AT) [9, 10] in diabetes. Non-enzymatic glycosylation following persistent hyperglycaemia, a key characteristic of diabetes, results in an excessive accumulation of advanced glycosylation end-products (AGEs) in most human organs and tissues [11]. This includes, but is not limited to, muscles, nerves, skin and tendons [12, 13]. Concomitant remodelling in the structural composition of the affected tissue [14] could alter mechanical behaviour, and in the presence of other established risk factors such as diabetes-related peripheral neuropathy (DPN) and foot deformity [4], may increase the risk of DFU development.

Increased tissue stiffness [15], particularly in the AT [16], and decreased tissue thickness are perceived to be independent risk factors for DFU. Increased plantar tissue stiffness (PTS) is thought to alter the distribution of tensile stresses in the plantar soft tissues during gait [15]. Consequently, repetitive biomechanical stresses normally sustained by the foot through activities of daily living may no longer be as effectively dissipated. Combined with a reduction in plantar tissue thickness (PTT), these changes could collectively decrease the mechanical loads required to initiate soft tissue breakdown and thus lead to DFU formation.

As a separate entity, increased Achilles tendon stiffness (ATS) reduces ankle joint motion and is associated with increased forefoot plantar pressures [16]. The AT is a compliant tendon, and its ability to stretch and recoil as it produces propulsion and absorbs ground reaction forces is integral to locomotion [17]. Increased ATS could negatively affect its dynamic potential and ankle joint range of motion [18]. Such changes may not only increase the AT’s susceptibility to mechanical fatigue [19], and thus predisposition to Achilles tendinopathy [20], but it could also lead to the abnormal distributions of forces across the plantar surface of the foot during weightbearing activities. Should these forces be concentrated over a focal area, plantar pressures could potentially increase beyond the withstanding capacity of affected plantar tissues.

Altogether, these changes bear the potential to reduce shock-absorbing capacity of the plantar tissues thus lowering the threshold for which integrity of the skin is breached, while at the same time magnify biomechanical stresses at localised sites, thereby considerably increasing the individual’s propensity to ulceration. However, the extent to which tissue thickness and stiffness contributes to DFU pathogenesis is unclear. Ulcerations and their recurrence are frequent sequelae [6, 21], yet prospective studies investigating changes to plantar tissue structure and function appear scarce. Therefore, the aim of this systematic review was to evaluate the evidence for changes to soft tissue thickness and stiffness at the plantar tissues and AT in people with diabetes.

## Methods

This systematic review was conducted in compliance with the Meta-analyses Of Observational Studies in Epidemiology (MOOSE) [22] and the Preferred Reporting Items for Systematic Reviews and Meta-Analyses (PRISMA) [23] guidelines. The protocol was prospectively registered on Prospero [CRD42020166614].

### Search strategy

Six electronic databases were systematically searched by the first reviewer (B.K.) for studies published from database inception until 1st October 2020. The databases were: EBSCOhost (AMED, CINAHL, MEDLINE), ProQuest (Health & Medical Collection, Nursing & Allied Health Database), and Web of Science. No date restrictions were imposed, however, studies were restricted to those published in English and in peer-reviewed journals only. Boolean operators and relevant MeSH terms were used and keywords were adapted for each database (Additional file 1).

### Inclusion and exclusion criteria

Studies which met criteria in the following five categories were eligible for inclusion. (1) Population: cohorts comprised of adults 18 years old and above were included; experiments conducted on animals, artificial tissues, cadavers or minors below 18 years old were excluded. (2) Interventions: no restrictions set. (3) Comparison: studies which had made a comparison between cohorts with and without diabetes were included; non-comparative studies, specifically studies which had not included a control group of otherwise healthy adults, were excluded. (4) Outcomes: in-vivo assessments of either thickness and/or stiffness of plantar soft tissues and/or the AT were included; in-vitro studies, studies which assessed other body parts or internal body organs, or technical studies solely evaluating the physics or engineering of the measurement technique were excluded. (5) Type of study: quantitative primary research studies published in peer-reviewed journals were included; qualitative studies, case reports, commentaries, conference proceedings, narrative reviews, and studies published in non-peer reviewed journals were excluded.

### Study selection

All articles retrieved in the search were first exported to RefWorks for removal of duplicates and subsequently exported to Covidence. Two reviewers (B.K. and R.B.) independently screened all titles and abstracts against pre-established inclusion and exclusion criteria. Articles which appeared to be eligible were retrieved in full and assessed by both reviewers. Upon mutual agreement for inclusion, the reference lists of included articles were additionally screened. This iterative process was performed until no further articles could be included. Where full-text articles could not be accessed, university library services were employed and authors contacted directly; this was successful on six occasions. Throughout this process, discrepancies were first resolved by consensus between the two reviewers (B.K. and R.B.) with adjudications sought from a third reviewer (J.W.) where required.

### Methodological quality assessment

All included studies were appraised for their methodological quality using a modified critical appraisal tool for quantitative studies developed by McMaster University [24]. This modified tool comprehensively evaluates 14 different aspects of methodological quality, categorised under the domains: study purpose, literature, design, sample, outcome, assessment technique, results, conclusions and implications. Quality assessment was performed by the first reviewer (B.K.) and checked in full by the second reviewer (R.B.). As the thresholds for distinguishing between study quality grades have not been established, study quality was scored upon mutual agreement by two reviewers (B.K. and R.B.). Assessment rubrics [25] provided by McMaster University to guide the completion and interpretation of each criterion were adhered to, and where additional criteria were applied by the reviewers, these are detailed as follows.

#### Criteria for assessing sample

The sample description was deemed as limited if participant inclusion or exclusion criteria, and/or baseline characteristics of body mass index (BMI), duration of diabetes or HbA1c levels of the cohort were not reported. This lack of detail limits the reviewers’ attempts to establish comparability of diabetes, DPN and DFU cohorts within studies. Potential confounders were identified a priori as age, sex and BMI due to their known effects on soft tissue thickness [26–28] and stiffness [29–32]. Where groups were matched for all three baseline demographics, studies were deemed to have adopted strategies to reduce confounding. Where studies matched for only one or two of the aforementioned baseline demographics or any other potential confounders, the confounding strategy was deemed as limited.

#### Criteria for assessing reliability or validity of outcome measures

Studies which had either tested for or cited previous works demonstrating the reliability or validity of their chosen assessment tool for the relevant outcome measures were recognised to have addressed these domains. Where reliability or validity was addressed for other assessment techniques within the study, but not those relating to PTT, PTS, Achilles tendon thickness (ATT) or ATS outcomes, or was not mentioned at all, the study was deemed to not have addressed this domain.

### Data extraction

Data extraction was performed independently by the first reviewer (B.K.) and checked for accuracy by the second reviewer (R.B.). A pre-specified data extraction sheet was used to extract data from all studies, which includes: (1) Article information: title, authors and year of publication; (2) Methods: study design, setting, participant demographics, participant inclusion and exclusion criteria, type of assessment tool utilised, outcome metric, and method of measurement for thickness or stiffness; and (3) Main findings. Within studies, statistical significance was defined as *p* < 0.05.

### Data synthesis

A descriptive synthesis was conducted using summary data from individual studies to identify the directionality of PTT, PTS, ATT and ATS changes in people with diabetes when compared to their controls. Absolute values for PTT and ATT were further extracted. Where sufficient data permits, subgroup analysis was undertaken to identify the presence of any anatomical variations across the plantar aspect of the foot and the length of the AT, and to explore how findings may compare across cohorts with different diabetes-related complications. This includes people with DPN or people with either an active or history of DFUs.

## Results

**Fig. 1:**
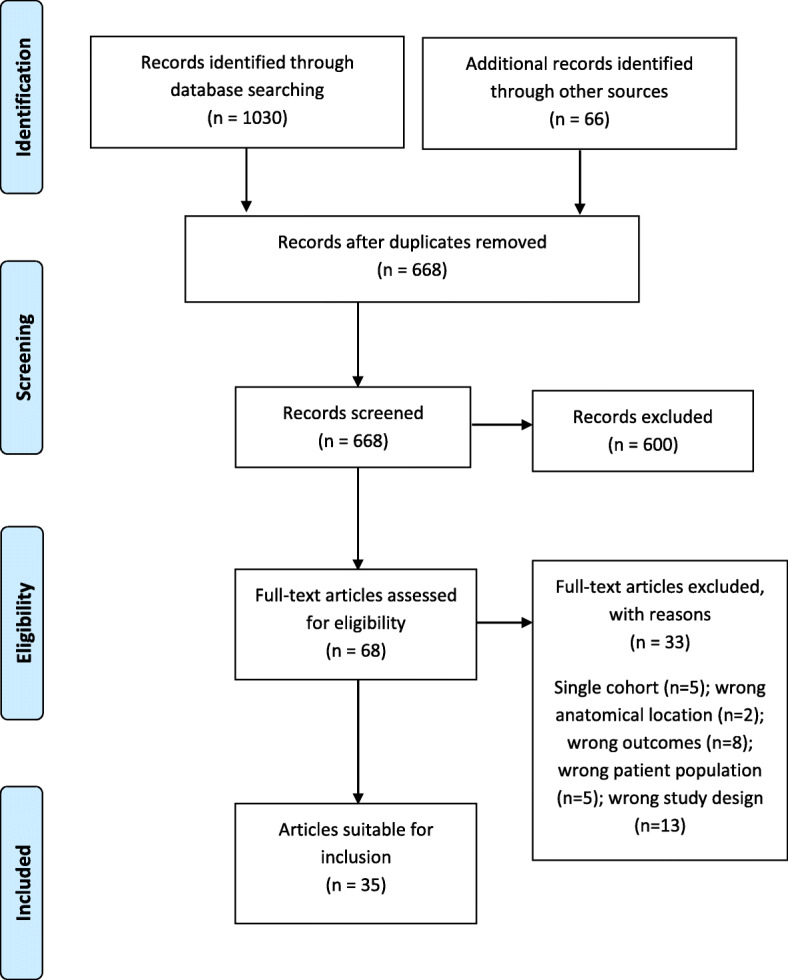
PRISMA diagram

The search strategy yielded a total of 668 articles, from which 68 full-text articles were screened (Fig. 1). 33 articles were excluded and the reasons for exclusion can be found in Additional file 2. A total of 35 studies were included in the analysis, giving a pooled diabetes sample of 1379 and 861 controls (Table 1). 21 studies [33–53] (60%) included people with Type 2 diabetes only, which is recognised to account for approximately 90–95% of diagnosed cases of diabetes worldwide [54].

**Table 1:** Overview of studies

No.	Author (year)	Study Design	Aim	Setting; Recruitment timeframe; Sample size and comparison groups	Parameters assessed and assessment tools utilised	Anatomical sites examined
**Plantar soft tissues**						
1	Kwak (2020) [34]	–	To compare the mechanical properties and stress-strain behaviours of plantar heel soft tissues between people with Type 2 diabetes, young and old control participants.	Foot and ankle tertiary referral clinic at the Seoul National University Bundang Hospital, Seoul, South Korea; Recruitment time-frame: – ■ 10 young ctrls ■ 10 older ctrls ■ 10 T2DM	PTS: Subject-specific finite element model	Heel
2	Lechner (2019) [36]	Descriptive, exploratory study	To compare the structure, function and molecular markers of dry and cracked foot skin between people with and without diabetes.	Department of Dermatology and allergy; Charité-Universitätsmedizin Berlin, Germany; September 2016 – April 2017; ■ 20 ctrls ■ 40 T2DM	PTT: Optical coherence tomography PTS: Cutometer	MTH 1, Heel
3	Oh (2018) [37]	Retrospective	To investigate the severity of pedal soft tissue atrophy caused by diabetes and aging.	Hospital, Daegu, South Korea; February 2009 – February 2016; ■ 47 ctrls ■ 52 T2DM	PTT: MRI	MTH 1, 2, 3, 4, 5
4	Kumar (2015) [39]	Observational	To evaluate the thickness of intrinsic foot muscles, plantar skin, plantar fascia and plantar fat pad in people with Type 2 diabetes (with and without DPN).	Tertiary hospital, Manipal, India; July 2013 – January 2014; ■ 30 ctrls ■ 12 T2DM ■ 18 T2-DPN	PTT: US	MTH 1, 2, 3, 4, 5
5	Chatzistergos (2014) [40]	–	To investigate the correlation between the mechanical properties of the heel pad of people with Type 2 diabetes and the clinical parameters used to monitor their health and ulceration risk (e.g. ABI, duration of diabetes, FBS, HbA1c, triglycerides and VPT)	Diabetic referral centre, Chennai, India; Recruitment time-frame: – ■ 17 ctrls ■ 35 T2DM	PTT and PTS: US probe connected with a dynamometer	Heel
6	Jan (2013) [42]	–	To investigate the effect of diabetes on the biomechanical properties and plantar pressure distribution in the pathogenesis of DFU.	Recruitment setting: – Recruitment timeframe: – ■ 6 ctrls ■ 7 T2-DPN	PTT and PTS: TUPS	MTH 1
7	Chao (2012) [43]	–	To evaluate the association between skin blood flow and oedema and epidermal thickness in the feet of people with and without diabetes.	Diabetic clinic of a local hospital, Hong Kong; January 2009 – November 2010; ■ 33 ctrls ■ 35 T2DM ■ 19 T2-DPN with DFU (active or a history of DFU)	PTT: US	Hallux
8	Periyasamy (2012) [44]	Pilot study	To investigate the feasibility of measuring PTS variation in people with diabetes.	Outpatient clinics of Biomedical and Endocrinology Lab, All India Institute of Medical Sciences, New Delhi, India; January – March 2011; ■ 10 ctrls ■ 10 T2DM ■ 10 T2-DPN	PTS: Durometer	Hallux, MTH 1, 2, 3–5, midfoot, heel
9	Chao (2011) [45]	–	To examine the changes in epidermal thickness and biomechanical properties of plantar soft tissue in people with Type 2 diabetes (with and without either DPN or DFU).	Diabetic clinic of a local hospital, Hong Kong; Recruitment time-frame: – ■ 40 ctrls ■ 34 T2DM ■ 32 T2-DPN ■ 16 T2-DFU (active or a history of)	PTT: US (epidermis only); TUPS (total plantar soft tissue thickness) PTS: TUPS (total plantar soft tissue stiffness)	Hallux, MTH 1, 3, 5, heel
10	Sun (2011) [55]	Cross-sectional study	To compare the thickness and stiffness of plantar soft tissues between people with DPN and healthy participants.	University research laboratory, Hong Kong; Participants recruited from a local outpatient diabetes clinic; Recruitment time-frame: – ■ 54 ctrls ■ 70 DPN (early-stage)	PTT and PTS: TUPS	Hallux, MTH 1, 2, heel
11	Hsu (2009) [46]	–	To compare micro- and macrochambers mechanical properties between people with Type 2 diabetes and age-matched healthy participants.	Tertiary care hospital, Taiwan; Recruitment time-frame: – ■ 16 ctrls ■ 18 T2DM (2 with DPN; all nil history of DFUs)	PTT and PTS: US with a load cell	Heel
12	Hsu (2007) [49]	Pilot study	To compare the energy dissipation ratio in the plantar soft tissue under the metatarsal heads between people with Type 2 diabetes and age-matched healthy participants.	Recruitment setting: – Recruitment timeframe: – ■ 8 ctrls ■ 13 T2DM (nil with DPN)	PTT: US with a load cell	MTH 1, 2, 3, 4, 5
13	Cheung (2006) [62]	Preliminary / pilot study	To test the feasibility of applying magnetic resonance elastography to map the elastic modulus of the plantar fat pads, in participants with and without diabetes.	Department of Radiology, Dartmouth Medical Centre, Lebanon; Recruitment timeframe: – ■ 12 ctrls† ■ 4 DPN	PTS: Magnetic resonance elastography	Heel
14	Hashmi (2006) [50]	Investigative report	To quantify specific glycation products generated in plantar epidermal proteins in individuals with Type 2 diabetes and age-matched controls, and to compare these data with the viscoelastic properties of the epidermis.	Diabetes Unit and the Diabetes Eye Screening Unit at Whittington hospital, London, UK; Recruitment timeframe: – ■ 87 ctrls ■ 103 T2DM	PTT: US PTS: Cutometer	MTH 3
15	Puri (2005) [51]	–	To examine changes in tissue echogenicity due to the altered material properties of the plantar foot in diabetes.	Recruitment setting: – Recruitment timeframe: – ■ 8 ctrls ■ 16 T2DM (3 with neuropathic DFUs)	PTS: Durometer	Hallux, MTH 1, 3–5, medial and lateral heel
16	Mueller (2003) [56]	–	To determine the primary forefoot structural factors which predict regional PPP during walking in groups of people with and without DPN.	Multidisciplinary tertiary-care diabetic foot clinic and the database from the Institution Diabetes Research Training Centre, Washington University School of Medicine, St. Louis, USA; Recruitment timeframe: – ■ 20 ctrls ■ 20 DPN with a history of DFUs	PTT and PTS: SXCT	PTT: MTH 1, 2, 3, 4, 5 PTS: MTH 1, 3, 5
17	Thomas (2003) [52]	Preliminary study	To identify relationships between foot pressure, tissue stiffness and thickness at different severities of DPN.	Recruitment setting: – Recruitment timeframe: – ■ 9 ctrls ■ 18 T2-DPN (5 with active DFU).	PTT: US PTS: Durometer	Hallux, MTH 2, 3–5, heel
18	Klaesner (2002) [63]	Case-control study	To determine if a difference exists in the plantar soft tissues of people with DPN compared with age-matched controls.	Data collection performed in an academic physical therapy laboratory; Participants recruited from multiple sources including those who had participated in previous studies in the laboratory, Washington University’s volunteers for Health subject database, the Diabetic Foot Centre at Barnes-Jewish Hospital, and physician referral, St. Louis, USA; Recruitment timeframe: – ■ 20 ctrls ■ 20 DPN with a history of DFU	PTS: Indentor system;	MTH 1, 3, 5, heel
19	Robertson (2002) [57]	–	To investigate relationships between structural changes of the forefoot in people with diabetes with a prior plantar DFU and in matched controls.	Multi-disciplinary tertiary-care diabetes clinic, Washington University School of Medicine, St. Louis, USA; Recruitment timeframe: – ■ 16 ctrls ■ 16 DPN with a history of DFU	PTT: Computed tomography	MTH 1–5
20	Hsu (2000) [53]	–	To compare the heel-pad mechanical properties in people with Type 2 diabetes (with and without forefoot DFU)and age-matched healthy participants using a specially designed loading-unloading device.	Recruitment setting: – Recruitment timeframe: – ■ 20 ctrls ■ 21 T2DM (38% with DPN) ■ 12 T2-DFU (all active, Wagner grade 2 or 3; 75% with DPN)	PTT and PTS: US with a loading/unloading device	Heel
21	Zheng (2000) [58]	–	To investigate the biomechanical properties of plantar tissues between older participants with DPN and healthy younger participants.	Recruitment setting: – Recruitment timeframe: – ■ 4 young ctrls ■ 4 elderly DPN	PTT and PTS: US indentation system	Hallux, MTH 1, 2, heel
22	Piaggesi (1999) [64]	–	To investigate if neuropathy-associated modification of skin elasticity is found before the occurrence of DFU.	Outpatient diabetic clinic, Pisa, Italy; June – December 1996; ■ 36 ctrls ■ 36 DM ■ 36 DPN	PTS: Durometer	Midfoot (median and lateral), heel, posterior mid-calf (as a control site)
23	Brink (1995) [59]	–	To investigate potential differences in periarticular soft tissues at the plantar pedis between people without diabetes and people with a history of neuropathic DFUs.	Recruitment setting: – Recruitment timeframe: – ■ 15 young ctrls ■ 10 older ctrls ■ 10 DPN with a history of recurrent DFUs	PTT: US PTS: Durometer	MTH 1, 2, 3, 4, 5, heel
24	Gooding (1986) [60]	–	To quantify the loss of foot pad thickness and investigate its relationship to ulceration of the foot.	Recruitment setting: – Recruitment timeframe: – ■ 24 ctrls ■ 38 DM ■ 11 DFUs (active or a history of)	PTT: US	MTH 1, 2, 3, 4, 5, heel
25	Gooding (1985) [61]	–	To investigate whether it is feasible for US to evaluate heel pad thickness without the use of radiation.	Recruitment setting: – Recruitment timeframe: – ■ 10 ctrls ■ 38 DM	PTT: US	Heel
**Plantar soft tissues and Achilles tendon**						
26	Cheing (2013) [41]	–	To compare the biomechanical properties of the ankle–foot complex of people with diabetes (with and without DPN) with healthy individuals, and to examine its correlation with postural control.	Two local outpatient diabetes clinics, Hong Kong; Recruitment timeframe: – ■ 32 ctrls ■ 23 T2DM ■ 9 T2-DPN	PTT, PTS, ATT and ATS: TUPS	Hallux, MTH 1, 3, 5, heel; AT: Distal portion
**Achilles tendon**						
27	Harish (2020) [33]	–	To evaluate sonographic changes in the AT of people with Type 2 diabetes including thickening, hypoechogenicity, loss of fibrillary pattern and alterations in the elasticity of the AT.	Recruitment setting: – Recruitment timeframe: – ■ 61 ctrls ■ 81 T2DM (30 with symptoms suggestive of DPN, 7 with active DFUs, 8 with leg amputations)	ATT: US ATS: Elastography (Shear wave elasticity imaging)	ATT: Proximal, mid- and distal portions ATS: Distal portion only
28	İyidir (2019) [35]	Cross-sectional study	To evaluate the elastographic features of AT with Acoustic Radiation Force Impulse in people with and without DPN.	Endocrinology and radiology departments of Başkent University, Ankara, Turkey; March 2016 – July 2017; ■ 30 ctrls ■ 23 T2DM ■ 22 T2-DPN	ATT: US ATS: Elastography (Acoustic Radiation Force Impulse)	Mid-portion
29	Couppé (2016) [68]	Cross-sectional study	To compare the effect of glycaemic control (based on 2-yr average HbA1c) in two groups of men with diabetes (Type 1 and Type 2) and either well or poorly controlled diabetes.	Recruitment setting: – Recruitment timeframe: – ■ 11 ctrls ■ 44 DM (22 well-controlled DM, 22 poorly-controlled DM)	ATS: US Collagen tendon fibril density: Electron microscopy Collagen cross-links: Biopsy specimens	Distal
30	Evranos (2015) [38]	Cross-sectional study	To evaluate ATT and ATS in people with Type 2 diabetes (with and without foot disease) and to investigate the factors that influence these.	Study conducted at endocrinology and radiology departments of a university hospital; Subjects recruited from diabetes clinics, Ankara, Turkey; July 2012 – December 2014; ■ 33 ctrls ■ 43 T2DM ■ 35 T2-DFU	ATT: US ATS: Elastography (strain)	Proximal, mid- and distal portions
31	Papanas (2009) [47]	–	To study AT morphology on MRI in people with Type 2 diabetes (with and without DPN).	Outpatient clinic of the diabetic foot, tertiary care setting, Greece; Recruitment timeframe: – ■ 16 ctrls ■ 19 T2DM ■ 19 T2-DPN	ATT and AT volume: MRI	–
32	Batista (2008) [65]	–	To identify any inherent structural pathology in a consecutive group of asymptomatic individuals with diabetes that might be associated with increased stiffness and the development of forefoot DFUs.	Department of Orthopaedic Surgery at the Federal University of São Paulo, Brazil, and Orthopaedic Surgery Clinic at Carmino Caricchio Hospital, Brazil; Recruitment timeframe: – ■ 10 ctrls ■ 60 DM	ATT: US Morphology: US	–
33	Akturk (2007) [48]	–	To investigate the effect of diabetes on the AT that may contribute to the long-term complications in the foot-ankle complex and to investigate the factors relating to its thickening.	Endocrinology clinic; Recruitment timeframe: – ■ 34 ctrls ■ 55 T2DM	ATT: US	Mid-portion
34	D’Ambrogi (2005) [66]	–	To examine foot function in the presence of diabetes-induced alterations of the anatomical and biomechanical unit formed by the AT, plantar fascia and MTPJs.	Outpatient clinics of the Metabolic Diseases Department at the University of Rome “Tor Vergata”, Italy; Recruitment timeframe: – ■ 21 ctrls ■ 27 DM ■ 19 DPN ■ 15 DPN with a history of DFU (up to 3 months)	ATT: US	Proximal, mid- and distal portions
35	Giacomozzi (2005) [67]	–	To examine the effects that diabetes-induced alterations of AT, plantar fascia and 1st MTPJ — both anatomical and functional — may have on foot loading.	Outpatient clinics of the Metabolic Diseases Department at the University of Rome “Tor Vergata”, Italy; Recruitment timeframe: – ■ 21 ctrls ■ 27 DM ■ 19 DPN ■ 15 DPN with a history of DFU (up to 3 months)	ATT: US	Proximal, mid- and distal portions

Symbols: – Not stated, † Discrepancy identified in-text

*Abbreviations*: *ABI* ankle brachial index, *AT* achilles tendon, *ATT* achilles tendon thickness, *ATS* achilles tendon stiffness, *Ctrls* control group, *DFU* diabetes-related foot ulcer, *DM* diabetes mellitus, *DPN* diabetes-related peripheral neuropathy, *FBS* fasting blood sugar, *MRI* magnetic resonance imaging, *MTH* metatarsal head (plantar), *MTPJ* metatarsophalangeal joint, *SXCT* spiral X-ray computed tomography, *T2* type 2 diabetes only, *TUPS* tissue ultrasound palpation system, *PTT* plantar tissue thickness, *PTS* plantar tissue stiffness, *US* ultrasonography, *VPT* vibration perception threshold

Studies were published across three decades (1985 to 2020), with sample sizes ranging from 8 to 190 participants. The mean age of participants with diabetes was 58.5 years and their mean BMI was 27.3. The mean age of controls was 51.6 years and their mean BMI was 26.0. Participant comparison groups included healthy controls, people with diabetes but without DPN, people with DPN, and people with either an active or history of DFUs.

From 35 included studies, 20 studies evaluated PTT outcomes [36, 37, 39–43, 45, 46, 49, 50, 52, 53, 55–61], 19 studies evaluated PTS outcomes [34, 36, 40–42, 44–46, 50–53, 55, 56, 58, 59, 62–64], 9 studies evaluated ATT outcomes [33, 35, 38, 41, 47, 48, 65–67] and 5 studies evaluated ATS outcomes [33, 35, 38, 41, 68]. Heterogeneity among methods of measuring thickness and stiffness outcome metrics was observed which prohibited direct comparison across studies and precludes the possibility of conducting a meta-analysis.

### Methodological quality assessment

All 35 papers included were non-randomised observational studies assessed to be of low quality. Overall, the degree of reporting was deemed to be inadequate for the following components of methodological quality: study design, sample size justifications, strategies to reduce confounding, reliability and validity of outcome measures utilised, and assessment techniques (Table 2).

**Table 2:**
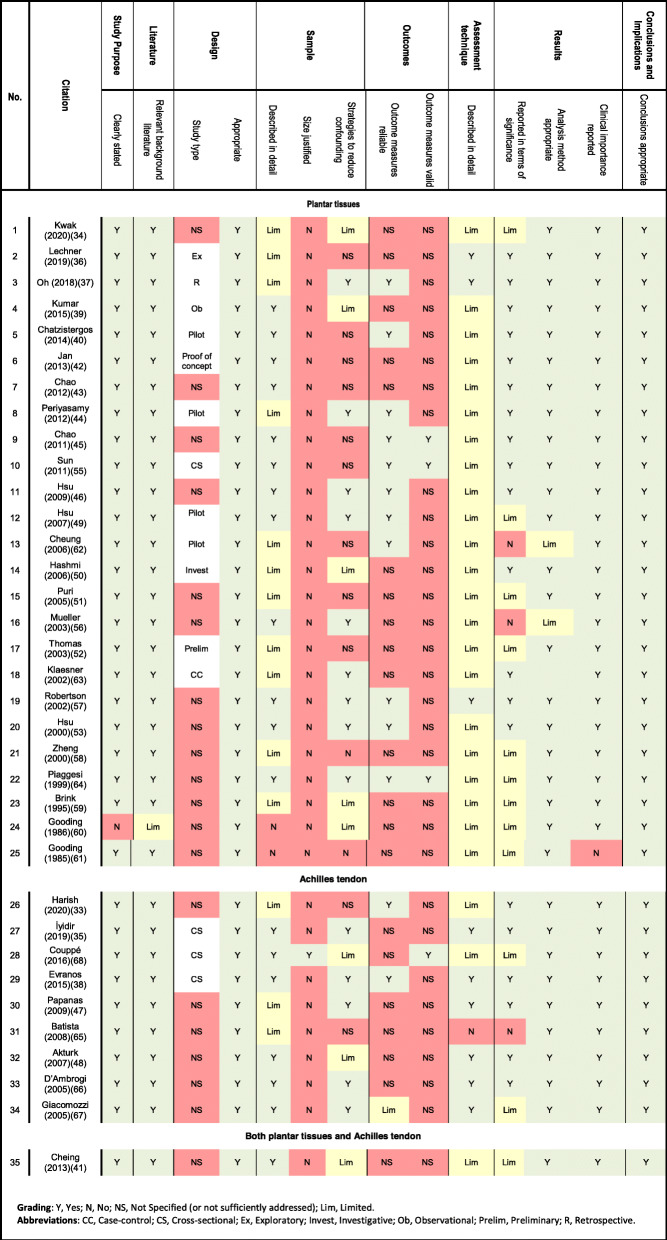
Methodological quality appraisal

#### Study purpose, literature and design

The purpose of the study and supporting background literature was generally well reported. Even though study designs were explicitly stated in only 43% of studies (15/35), the designs were considered appropriate.

#### Sample

Consecutive [47, 65] or convenience [41, 43, 45, 55] sampling was reported in 17% of studies (6/35); the remaining 83% of studies (29/35) made no mention of their sampling method. Sample size was justified with power calculations in only one study (3%) [68]. The difference between groups with diabetes and control groups in age, sex and BMI was statistically not significant in 40% of studies (14/35) [35, 37, 38, 44, 46, 47, 49, 53, 56, 57, 63, 64, 66, 67].

#### Reliability and validity of outcome measures

Reliability of outcomes measures was reported in 40% of studies (14/35). For PTT, reliability was reported for computed tomography (CT) [57], magnetic resonance imaging (MRI) [37], ultrasonography (US) probe with dynamometer [40], tissue ultrasound palpation system (TUPS) [45, 55] and US with a load cell or a loading/unloading device [46, 49, 53]. For PTS, reliability was reported for durometer [44, 64], magnetic resonance elastography [62], TUPS [45, 55], US probe with dynamometer [40] and US with a load cell or a loading/unloading device [46, 53]. For ATS, reliability was reported for shear wave elasticity imaging [33], strain elastography [38] and US [68]. None of the studies that assessed ATT reported on the reliability of their assessment technique.

Within these studies, a coefficient of variance between repeated measurements was found to be less than or equal to 5% in six studies [40, 46, 49, 53, 57, 68] and less than 10% in one study [62]. Excellent intra-class correlation coefficient (ICC) of the test-retest reliability was found in two studies (ICC > 0.8) [33, 37]. Intra-operator reliability was tested but not reported in one study [67]. No mention of reliability was made in the remaining 60% of studies (21/35).

Validity of outcome measures was reported in 11% of studies (4/35). All four studies had cited previous works in demonstrating the validity of their chosen assessment tools for their specified outcome measure(s). Two studies [45, 55] have indicated that the validity of the TUPS had been demonstrated for both PTT and PTS. One study [44] had indicated that the validity of the durometer had been demonstrated for PTS. In relation to the AT, only one study [68] had indicated that the validity of US had been demonstrated for ATS. None of the studies that assessed ATT had reported on the validity of their assessment technique. No mention of validity was reported in the remaining 89% of studies (31/35).

### Outcome metrics

For PTS, 18 different outcome metrics were adopted across 19 studies (Table 3). Of these, two or more metrics were utilised by six studies. For ATS, four different outcome metrics were noted across five studies. These include an elastographic colour map [38], shear wave velocity [33, 35], Young’s modulus [41], and a measurement of the slope over the last 20% of tendon deformation from fitting the force-deformation data to a third-order polynomial [68].

**Table 3:** Plantar tissue stiffness outcome metrics

No.	Outcome metrics	Author
1	Compressibility index	Hsu (2000) [53]
2	Elasticity (Ur/Uf) (%)	Lechner (2019) [36]
3	Total deformation (Uf) (mm)	Lechner (2019) [36]
4	Elastic modulus	Hsu (2009) [46]; Hsu (2000) [53]
5	Effective Young’s modulus (kPa)	Cheing (2013) [41]; Jan (2013) [42]; Chao (2011) [45]; Zheng (2000) [58]
6	Young’s modulus (kPa)	Sun (2011) [55]
7	Initial modulus (kPa)	Jan (2013) [42]
8	Non-linear modulus (kPa)	Jan (2013) [42]
9	Initial shear modulus (MPa)	Kwak (2020) [34]
10	Shear modulus (kPa)	Cheung (2006) [62]
11	Strain-hardening exponent	Kwak (2020) [34]
12	Ratio of the final displacement reading at 120s to the maximum displacement at 60s (where there is an application of stress for the first 60s, followed by 60s without)	Hashmi (2006) [50]
13	Change in force divided by the change in displacement, which was then transformed using a parallel three-element viscoelastic model developed by the authors	Mueller (2003) [56]
14	Change in force divided by the change in displacement, which was then transformed using a parallel three-element viscoelastic model developed by the authors; where, K_1_ = entire range of indentation; K_2_ = second portion of the indentation curve.	Klaesner (2002) [63]
15	Slope of the final part of the force/deformation curve	Chatzistergos (2014) [40]
16	Shore degrees (°)	Periyasamy (2012) [44]; Puri (2005) [51]; Thomas (2003) [52];
17	Shore indices	Periyasamy (2012) [44]
18	Shore A values	Brink (1995) [59]

### Assessment techniques

#### Assessment tools

##### Plantar tissue thickness

From 20 studies investigating PTT, five different assessment tools were used. 80% of studies (16/20) utilised US-based measures [39–43, 45, 46, 49, 50, 52, 53, 55, 58–61], while the following instruments were used by one study each: CT [57], MRI [37], optical coherence tomography [36] and spiral X-ray CT [56]. Of US-based studies, 50% (8/16) utilised US alone [39, 43, 45, 50, 52, 59–61], 25% utilised TUPS [41, 42, 45, 55], 25% combined use of US with either a load cell or indentation system [46, 49, 53, 58] and one study utilised an US connected with a dynamometer [40]. Chao et al. (2011) [45] was the only study to utilise two different techniques in their assessment: US for the epidermis only and TUPS for total PTT. 63% of studies (10/16) utilised B-mode US [39, 40, 43, 45, 46, 49, 52, 55, 60, 61], 19% utilised M-mode US [42, 45, 49], 13% utilised A-mode US [45, 50] and 19% did not specify the US modality utilised [41, 58, 59]; Chao et al. (2011) [45] and Hsu et al. (2007) [49] used more than one US modality.

##### Plantar tissue stiffness

From 19 papers assessing PTS, six different assessment tools were used. 47% of studies (9/19) utilised US-based measures [40–42, 45, 46, 53, 55, 58, 62], 26% utilised the durometer [44, 51, 52, 59, 64], 11% utilised cutometer [36, 50], and the following instruments were used by one study each: indentor system [63], subject-specific finite element model [34] and spiral X-ray CT [56]. Of US-based studies, 44% (4/9) utilised TUPS [41, 42, 45, 55], 22% combined use of the US with either an indentation system or load cell [46, 58], and one study each utilised: US alone [53], US connected with a dynamometer [40] and magnetic resonance elastography [62]. Within these, 50% utilised B-mode US [40, 46, 53, 55], 25% utilised M-mode US [42, 45], 13% utilised A-mode US [45] and 25% did not specify the US modality utilised [41, 58]; Chao et al. (2011) [45] used more than one US modality.

##### Achilles tendon thickness and stiffness

From nine studies assessing ATT, two different assessment tools are used. 89% of studies (8/9) utilised US-based measures, with 78% utilised US [33, 35, 38, 48, 65–67] and 11% utilising TUPS [41]. The remaining study utilised the MRI [47]. From five studies assessing ATS, all five studies utilised US-based tools albeit using five different assessment techniques. One study each utilised the acoustic radiation force impulse elastography [35], strain elastography [38], shear wave elasticity imaging [33], TUPS [41] and US [68].

#### Method of measurement

##### Plantar tissue thickness

PTT was measured as the distance between skin and bone in 70% of studies (14/20) [37, 41, 42, 45, 46, 49, 52, 53, 55–60]. A slight variation in measurement was noted in one study, with the authors measuring PTT as the distance between probe and bone instead [40]. Other heterogeneous methods of measuring PTT includes: measurements of the epidermis only [45, 50], the epidermis and upper dermis measured separately [43], the epidermis and dermis combined [39], and the epidermal-dermal junction zone only [36]. The method of measuring PTT was not explicitly stated in one study [61]. PTT values extracted from all 20 studies can be found in Additional file 3.

##### Achilles tendon thickness

Some uniformity was noted in the method of measurement for ATT across papers. ATT was measured as the anteroposterior diameter across the examined AT portion(s) in 56% of studies (5/9) [33, 35, 38, 47, 48]. However, these measurements were made in the longitudinal plane in two studies [35, 48] and from transverse scans in two other studies [33, 38]. The plane of measurement was not stated in Papanas et al’s (2009) study [47]. Instead of measuring the thickest point of the examined portion(s), Cheing et al. (2013) [41] was the only study to have adopted a different method of measurement. In their study, ATT was measured from the superior surface of the AT to its insertion at the posterior calcaneus. The method of measuring ATT was not explicitly stated in the remaining three studies [65–67]. ATT values extracted from all 9 studies can be found in Additional file 4.

#### Anatomic sites examined

##### Plantar tissue thickness

Across 20 studies examining PTT, 30% examined the hallux, 60% examined the first metatarsal head (MTH), 45% examined the second MTH, 45% examined the third MTH, 30% examined the fourth MTH, 40% examined the fifth MTH and 60% examined the heel. Robertson et al. (2002) [57] did not report their findings for each MTH individually, instead providing it as a mean and standard deviation across all five MTHs for each cohort. Thomas et al. (2003) [52] had similarly merged their findings for the third to fifth MTHs. None of the studies had examined PTT at the lesser toes.

##### Plantar tissue stiffness

Across 19 studies examining PTS, 37% examined the hallux, 58% examined the first MTH, 26% examined the second MTH, 32% examined the third MTH, 5% examined the fourth MTH, 26% examined the fifth MTH, 11% examined the medial and lateral midfoot, and 84% examined the heel. 16% of studies had merged their findings for the third to fifth MTHs [44, 51, 52]. One study had compared the microchambers and macrochambers in the heel [46]. One study had compared the plantar skin and fat layer at the heel [34]. None of the studies had examined PTS at the lesser toes.

##### Achilles tendon thickness and stiffness

Across 10 studies which had investigated either ATT and/or ATS, 20% of studies (2/10) had differentiated their measurements between all three portions of the AT. Both studies [33, 38] concurred in their method of measurement, in that the proximal third portion was measured at the myotendinous junction, the mid-portion was measured 2–6 cm above its insertion at the calcaneus, while the distal third portion was measured at the AT insertion at the calcaneus. Of the remaining studies, 60% [35, 41, 48, 66–68] examined only one out of three portions of the AT, while 20% [47, 65] had not specified the portion(s) they had examined.

### Key findings

The main findings for PTT, PTS, ATT and ATS outcomes are tabulated in Table 4.

**Table 4:** Main findings

Findings	Plantar tissue thickness	Plantar tissue stiffness	Achilles tendon thickness	Achilles tendon stiffness
**People with diabetes compared to healthy controls**				
Total no. of studies	20	19	9	5
↑	5% (1/20)	47% (9/19)	45% (4/9)	0
↓	10% (2/20)	0	0	20% (1/5)
NS	55% (11/20)	21% (4/19)	33% (3/9)	60% (3/5)
Other	30% (6/20)	32% (6/19)	22% (2/9)	20% (1/5)
**People with diabetes-related peripheral neuropathy compared to healthy controls**				
Total no. of studies	7	9	6	3
↑	0	78% (7/9)	83% (5/6)	0
↓	29% (2/7)	0	0	67% (2/3)
NS	57% (4/7)	22% (2/9)	17% (1/6)	33% (1/3)
Other	14% (1/7)	0	0	0
**People with an active or a history of diabetes-related foot ulcerations compared to healthy controls**				
Total no. of studies	8	6	3	1
↑	13% (1/8)	17% (1/6)	100% (3/3)	0
↓	0	0	0	0
NS	50% (4/8)	17% (1/6)	0	0
Other	37% (3/8)	66% (4/6)	0	100% (1/1)

Symbols: ↑ Significantly increased; ↓ Significantly decreased

*Abbreviations*: *NS* no significant differences

#### Plantar tissue thickness and stiffness

Across 20 studies examining PTT, no significant differences were found between people with and without diabetes across all examined anatomical site(s) in 55% of studies (11/20) [36, 40–42, 46, 49, 53, 55–57, 59] (Additional file 5). When compared with otherwise healthy controls, 10% of studies (2/20) reported significantly reduced PTT [39, 58] and 5% (1/20) reported significantly increased PTT in their cohort with diabetes [61]. Extensive variations were noted in the remaining 30% of studies (6/20), with plantar tissues observed to be significantly thicker, thinner and not significantly different between diabetes and healthy control groups. These findings further varied across anatomical sites examined [37, 43, 52, 60] or sub-groups [43, 45, 50] compared (Additional file 5).

Across 19 studies examining PTS, a significant increase in PTS for people with diabetes was observed in 47% of studies (9/19). Of these, 26% of studies (5/19) noted significantly increased PTS across all examined site(s) in cohorts with diabetes when compared to their healthy controls [40, 42, 45, 55, 58] and 21% of studies (4/19) shared this trend of significant increase across all bar one of their examined sites [36, 41, 44, 51] (Additional file 5). One study (5%) noted an increase in PTS across all examined sites, however it is uncertain if this reached significance as inferential statistics was not conducted for PTS differences between groups [56]. No significant differences in all examined sites between groups were observed in 21% of studies (4/19) [46, 53, 62, 64]. By contrast, none of the studies concluded that a reduction in PTS was found for their cohorts with diabetes. The remaining 26% of studies (5/19) reported variations in their findings depending on the anatomical region examined [34, 59], outcome metric utilised [50, 63] or sub-groups compared [52] (Additional file 5).

#### Achilles tendon thickness and stiffness

Across nine studies that analysed ATT, 44% (4/9) found a significant increase across all examined portion(s) in people with diabetes when compared to healthy controls [33, 35, 41, 66] (Additional file 6). No significant differences in ATT between groups were observed in 33% of studies (3/9) [38, 47, 67]. One (11%) study found reduced ATT in people with diabetes, however it is not known if this difference was significant as inferential statistics was not conducted for ATT measurements [65]. Sex variations in findings were noted in 22% of studies (2/9) [47, 48]. Both studies reported significantly greater ATT in women with Type 2 diabetes when compared to women without diabetes, but no significant differences were found between men with Type 2 diabetes and men without diabetes.

Across five studies that measured ATS, 60% (3/5) found no significant differences across all examined portion(s) between people with and without diabetes [35, 38, 41]. One study (20%) found a significant reduction in ATS for their cohort with diabetes [33] and the remaining study’s (20%) findings differed depending on the outcome metric utilised [68] (Additional file 6).

#### Sub-group analysis

##### Participants with diabetes-related peripheral neuropathy

Seven studies investigating PTT [39, 41, 42, 45, 52, 55, 58], nine studies investigating PTS [41, 42, 44, 45, 52, 55, 58, 62, 64], six studies investigating ATT [33, 35, 41, 47, 66, 67] and three studies investigating ATS [33, 35, 41] analysed their findings separately for participants with DPN. More than half of studies found no significant differences in PTT between people with DPN and healthy controls [41, 42, 52, 55] (57%, 4/7 studies) and significantly increased PTS in people with DPN compared to healthy controls [42, 45, 55, 58, 64] (56%, 5/9 studies). A further 22% studies [41, 44] (2/9) found significantly increased PTS across all bar one site for participants with DPN. The majority of studies found significantly increased ATT [33, 35, 41, 66, 67] (83%, 5/6 studies) and significantly reduced ATS (67%, 2/3 studies) in people with DPN when compared to healthy controls [33, 35]. Of these studies, the confounding effect of age could not be eliminated for Zheng et al. (2000) [58] as they had compared elderly DPN with young healthy controls.

##### Participants with an active or a history of diabetes-related foot ulcerations

Eight studies investigating PTT [43, 45, 52, 53, 56, 57, 59, 60], six studies investigating PTS [45, 52, 53, 56, 59, 63], three studies investigating ATT [38, 66, 67] and one study investigating ATS [38] analysed their findings separately for people with either an active or a history of DFUs. Half of studies investigating PTT (4/8) found no significant differences between groups [53, 56, 57, 59]. No clear pattern was identified for PTS (Additional file 5). All studies investigating ATT [38, 66, 67] (3/3) found ATT to be significantly increased in their cohort with DFU. The only study investigating ATS found stiffness to be significantly lower in people with active DFUs when compared with people with Type 2 diabetes and healthy controls at the distal (insertional) and mid-portion only; no significant differences were found between groups at the proximal segment (myotendinous junction) [38].

Brink (1995) [59] and Thomas et al. (2003) [52] were the only two studies to directly compare their findings for either active or healed DFU sites with non-ulcerated sites. Both studies found PTS at DFU sites to be increased when compared to non-ulcerated sites. Hsu et al. (2000) [53] limited their cohort to participants with active forefoot DFUs (Wagner grade 2 or 3) whilst investigating PTS at the heel.

##### Comparing findings across diabetes-related foot complications

In studies which compared findings across subgroups with different diabetes-related foot complications, no significant differences in PTT were found between people with diabetes and those with DPN [39, 41], as well as between people with and without DFU [53]. No significant differences in ATT was found between people with diabetes and those with DPN [33, 35, 47, 66], while ATT was found to be significantly increased in participants with DFU compared to those without DFU [38, 66, 67].

#### Anatomical variations

##### Plantar soft tissues

Changes to plantar tissue sub-structures are seldom reported. Chao et al. (2012) [43] measured the differences in tissue thickness between the epidermis and upper dermis, while Chao et al. (2011) [45] measured the differences in thickness between the epidermis and total plantar tissues. Regional variations in PTT were observed in 30% of studies (6/20), with findings which further varied depending on the anatomical sites [37, 43, 52, 60] or sub-groups [43, 45, 50] compared (Additional file 5); no clear pattern emerged from this.

Regional variations in PTS were observed in only one out of all other examined sites in 26% of studies (5/19) [36, 41, 44, 51, 52]. More extensive regional variations in PTS were noted in only one study [59] (5%) with contradictory findings (Additional file 5). Kwak et al. (2020) [34] reported differences between plantar fat pad and skin stiffness, while Hsu et al. (2009) [46] reported differences in micro- and macrochamber stiffness in the heel pads.

##### Achilles tendon

Only two studies distinguished their findings between the three segments of the AT (myotendinous junction, mid-portion and insertion) [33, 38], both studies utilised US. While they shared the observation that ATT findings for each individual segment had not differed from each other [33, 38], Evranos et al. (2015) [38] noted that ATS differed in the proximal segment (myotendinous junction) when compared with the mid- and distal (insertional) segments (Additional file 6). Additionally, only two studies analysed the US-derived echotexture of the AT [33, 65]. Altered patterns were found in people with diabetes in the form of hypoechogenicity, loss of fibrillar pattern, calcification at the tendon insertion at the calcaneus and/or retrocalcaneal bursitis.

## Discussion

### Principal findings

This systematic review investigated the evidence for differences in plantar soft tissue and AT thickness and stiffness in people with and without diabetes. The general consensus among studies was that diabetes is associated with a significant increase in PTS, however the differences in PTT is not significant. Studies also found that diabetes is associated with a significant increase in ATT, however the differences in ATS is not significant.

### Methodological considerations

Extensive methodological heterogeneities and deficiencies was a key finding of this systematic review. Sample sizes were seldom justified (3%, 1/35). Additionally, reporting was generally inadequate across the domains of study designs and the reliability and validity of outcome measures. Case definition for participants with diabetes and subgroups formed on the presence and history of DFU or DPN were often absent or inadequately defined. Furthermore, the risk of selection bias cannot be eliminated in the majority of studies as only 6% of studies adopted a consecutive sampling method for recruiting people with diabetes; the remaining 94% of studies (33/35) made no mention of their sampling strategy for their cohorts with diabetes.

No gold standard assessment techniques have been established for tissue thickness and stiffness assessments and this was reflected in the diverse approaches reported in this review. Studies that employed novel biomedical technologies and tissue engineering outcomes are additionally not easily reproduced and are not well understood in clinical settings. Ultrasound-based techniques were favoured – utilised by 80% of studies (16/20) investigating PTT, 47% of studies (9/19) investigating PTS, 89% of studies (8/9) investigating ATT, and 100% of studies (5/5) investigating ATS – but standardisation in its usage is lacking. Ultrasound-based techniques are also known to be highly dependent on the knowledge, experience and skill of the operator [69]. Therefore, the extent to which outcomes have been reliably measured is important. However, no mention of reliability was made in 60% of studies (21/35).

Studies further differed in their measurement techniques. Using PTT for example, while the majority of studies (70%, 14/20) measured PTT as the distance between skin and bone, overall six different methods of measurements were observed. Combined with the use of different assessment techniques and examination of different anatomical sites, these wide-ranging methodological heterogeneities made it difficult to compare and synthesise results across studies. The lack of standardisation also renders derivation of normal and diagnostic cut-off values extremely challenging and hampers routine clinical utility for at-risk screening, detection and longitudinal monitoring.

### Role of plantar soft tissues thickness and stiffness in DFU

On the plantar aspect of the foot, areas at high risk of ulceration are the apices of the toes, metatarsal heads, medial aspect of the midfoot and the heel [4]. This systematic review observed that evidence for the differences in PTS is more consistent across included studies than PTT (Additional file 5). However, regional variations in either PTT or PTS appear modest. This implies that other risk factors such as tissue load, in the presence of structural tissue changes, may be more important for the development of DFU rather than either factor alone. These findings reinforce the multi-factorial nature of plantar DFU pathogenesis [5, 6] and the need for further mechanistic and outcome focused research.

The lifetime incidence of DFUs is estimated to be up to 35% [6]. DFU recurrence rates are also high, with the pooled global recurrence rates estimated in a meta-analysis to be 22.1% per person-year (95% CI, 19.0–25.2%) [21]. However, the extent to which plantar soft tissue changes contribute to either the development of primary ulcerations or its recurrence is little understood. No firm conclusions can be drawn from this review, as only 6% of studies (2/35) directly compared either active or healed DFU sites with never-ulcerated sites. Additionally, none of the included studies measured differences in PTT or PTS at the apices of the toes. Combining the paucity of studies primarily investigating these associations with the methodological shortfalls identified in existing studies, the clinical significance of tissue thickness or stiffness changes in its role in contributing towards plantar DFUs remains unclear.

### Role of Achilles tendon thickness and stiffness in DFU

Achilles tendon changes in diabetes are recognised to potentially alter ankle biomechanics [16], increase forefoot plantar pressures and contribute to tissue breakdown [70]. This systematic review identified that people with diabetes, particularly those with DPN and DFU, may have a thicker AT but reduced AT stiffness. The International Working Group on the Diabetic Foot (IWGDF) guidelines [71] recommend AT lengthening procedures to be considered as a potential management option for the prevention of DFU recurrence. It is not clear what the implications of these findings are for such procedures. The authors of this systematic review are unaware of studies that have specifically investigated the association of either ATT or ATS with AT lengthening procedures and this merits further investigation.

Thickening in tendons are often regarded as pathological clinically [17, 72]. While the site of localised thickening could be important, this review and the evidence synthesised does not elucidate. It is further uncertain if a thicker or thinner AT, stiffer or less stiff AT, presenting simply by virtue of its different thickness or stiffness when compared to an otherwise healthy cohort, can therefore be viewed as pathological in diabetes. To the best of the authors’ knowledge, the normative values of ATT or ATS have not been established in a cohort of people with diabetes, such that even in the absence of symptomology, said AT changes should therefore be regarded as tendinopathic.

Asymptomatic AT pathologies are common in athletic and general populations [73–76]. While AT structural changes do not always trigger tendon pain [77], this could arguably be more prevalent in people with diabetes due to masking from DPN. However, the lack of symptoms may not negate the potential for occult AT thickness or stiffness changes to persist in its alterations to gait. To this end, this systematic review contributes to this research gap by providing indicative ATT values from included studies (Additional file 4). The extensive heterogeneity in outcome metrics prevented similar consolidations in findings for ATS. Further work is required and advances in technology may afford greater insights into the ATT and ATS changes in diabetes.

### Potential confounders

It is further unclear to what extent baseline factors such as age, sex, BMI, duration of diabetes and glycaemic control are associated with soft tissue changes in diabetes. In this systematic review, the differences between groups with diabetes and control groups in age, sex and BMI were statistically not significant in 40% of studies which appears to reduce the potential for confounding. However, studies reviewed have documented poor correlations between age with PTT [37, 40], PTS [40] and ATT [33] differences; BMI with PTT and PTS differences [40]; duration of diabetes with PTT [40], PTS [40] and ATT [33] differences; as well as HbA1c levels with PTT [39, 40], PTS [40] and ATT [33] differences between people with and without diabetes. Strong correlations have however been found between triglyceride levels and PTS differences in the heel for people with diabetes [40]. Nonetheless, it was difficult to tease out whether soft tissue alterations were due to the adequacy of glycaemic control, chronicity of hyperglycaemia, hypertriglyceridemia, severity of DPN or other potential confounders in demographics. This requires careful attention in future studies.

### Strengths and limitations

This systematic review has several strengths. The review included all studies published to date on the topic, including a body of knowledge from the biomedical technology field where assessment techniques were utilised in cohorts of people with diabetes and associated risk factors for DFU. Extensive search strategies were employed with detailed, careful and critical assessment to identify risk of bias. The review is, however, limited by the methodological deficiencies of included studies. The critical appraisal tool by McMaster University [24] has also not been validated and the threshold for distinguishing between grades of methodological quality have not been established. Substantial methodological heterogeneities prevented the conduct of meta-analysis which further limits the review. Additionally, two papers with similar cohorts may have contributed to the observed heterogeneity. However, since no pooled analysis was undertaken, the effect on the precision of the estimated outcome is unclear.

## Conclusions

This systematic review found some preliminary evidence supporting differences in soft tissue properties in the feet of people with and without diabetes. However, uncertainties remain largely due to methodological heterogeneities and deficiencies. Tissue thickness and stiffness are putative risk factors for DFU and may be a viable treatment target. High-quality studies using standardised and validated assessment techniques in well-defined populations are required to advance our understanding and to determine more fully the role of soft tissue thickness or stiffness changes in the pathogenesis of DFU. Should soft tissue compromise be indicative of imminent tissue breakdown in the diabetic foot, this has the potential to aid clinical triage and inform the timely adoption of targeted preventive measures.

## Supplementary Information

**Additional file 1.** Database search strategy.

**Additional file 2.** Reasons for article exclusion.

**Additional file 3.** Plantar tissue thickness values.

**Additional file 4.** Achilles tendon thickness values.

**Additional file 5.** Directionality of findings for plantar soft tissues.

**Additional file 6.** Directionality of findings for Achilles tendon.

## Data Availability

All data generated or analysed during this study are included in this published article (and its supplementary information files).

## Abbreviations

AGEs: Advanced glycosylation end-products
AT: Achilles tendon
ATS: Achilles tendon stiffness
ATT: Achilles tendon thickness
BMI: Body mass index
CT: Computed tomography
DFU: Diabetes-related foot ulceration
DPN: Diabetes-related peripheral neuropathy
ICC: Intra-class correlation coefficient
MOOSE: Meta-analyses Of Observational Studies in Epidemiology
MRI: Magnetic resonance imaging
MTH: Metatarsal head
PRISMA: Preferred Reporting Items for Systematic Reviews and Meta-Analyses
PTS: Plantar tissue stiffness
PTT: Plantar tissue thickness
TUPS: Tissue ultrasound palpation system
US: Ultrasonography
