# Supplementary material for: Plantar soft tissues and Achilles tendon thickness and stiffness in people with diabetes: a systematic review
Source: J Foot Ankle Res. 2021 Apr 28;14:35. doi: 10.1186/s13047-021-00475-7 (PMC8080343; doi:10.1186/s13047-021-00475-7)
Supplement: Supplementary file 1 — Additional file 1. Database search strategy. [file 13047_2021_475_MOESM1_ESM.docx]

**Additional file 1: Database search strategy**

| 1. **CINAHL (EBSCOhost)** | | |
| --- | --- | --- |
| S26 | S3 AND S24 AND S25 | 129 |
| S25 | S11 OR S14 | 122,485 |
| S24 | S15 OR S16 OR S17 OR S18 OR S19 OR S20 OR S21 OR S22 OR S23 | 238,318 |
| S23 | strain | 55,418 |
| S22 | (MH "Elasticity") OR "elasticity" | 5,525 |
| S21 | hardness | 1,080 |
| S20 | stiffness | 14,800 |
| S19 | diameter | 26,306 |
| S18 | depth | 44,987 |
| S17 | (MH "Skinfold Thickness") OR "thickness" | 33,319 |
| S16 | (MH "Biomechanics") OR "biomechanical" | 29,310 |
| S15 | mechanical OR (MH "Stress, Mechanical") | 53,904 |
| S14 | S12 OR S13 | 4,506 |
| S13 | “calcaneal tendon” | 1,651 |
| S12 | (MH "Achilles Tendinopathy") OR (MH "Achilles Tendon") OR "Achilles" | 4,496 |
| S11 | S4 OR S5 OR S6 OR S7 OR S8 OR S9 OR S10 | 118,199 |
| S10 | subcutaneous | 19,655 |
| S9 | cutaneous | 17,024 |
| S8 | (MH "Dermis") OR "dermis" | 7,791 |
| S7 | (MH "Epidermis") OR "epidermis" | 1,783 |
| S6 | (MH "Skin") OR "skin" | 91,597 |
| S5 | plantar soft tissues | 11 |
| S4 | pedal soft tissues | 0 |
| S3 | S1 OR S2 | 7,775 |
| S2 | diab* feet” | 70 |
| S1 | (MH Diabetic Foot") OR "diab* foot” | 7,763 |

| 1. **MEDLINE (EBSCOhost)** | | |
| --- | --- | --- |
| S26 | S3 AND S24 AND S25 | 373 |
| S25 | S11 OR S14 | 831,849 |
| S24 | S15 OR S16 OR S17 OR S18 OR S19 OR S20 OR S21 OR S22 OR S23 | 1,642,283 |
| S23 | strain | 511,635 |
| S22 | (MH "Elasticity") OR "elasticity" OR (MH "Elastic Modulus") | 61,116 |
| S21 | (MH "Hardness") OR "hardness" | 21,449 |
| S20 | stiffness | 63,671 |
| S19 | depth | 193,700 |
| S18 | diameter | 248,008 |
| S17 | (MH "Skinfold Thickness") OR "thickness" | 212,228 |
| S16 | (MH "Biomechanical Phenomena") OR (MH "Tensile Strength") OR (MH "Mechanics") OR "biomechanical" | 147,880 |
| S15 | (MH "Stress, Mechanical") OR "mechanical" | 430,279 |
| S14 | S12 OR S13 | 11,377 |
| S13 | “calcaneal tendon” | 4,907 |
| S12 | (MH "Achilles Tendon") OR "Achilles" | 11,279 |
| S11 | S4 OR S5 OR S6 OR S7 OR S8 OR S9 OR S10 | 821,252 |
| S10 | (MH "Subcutaneous Tissue") OR "subcutaneous" | 132,506 |
| S9 | cutaneous | 151,537 |
| S8 | (MH "Dermis") OR "dermis" | 96,847 |
| S7 | (MH "Epidermis") OR "epidermis" | 46,775 |
| S6 | (MH "Skin") OR "skin" | 627,778 |
| S5 | pedal soft tissues | 0 |
| S4 | plantar soft tissues | 34 |
| S3 | S1 OR S2 | 9,822 |
| S2 | diab* feet | 162 |
| S1 | (MH "Diabetic Foot") OR "diab* foot” | 9,785 |

| 1. **AMED (EBSCOhost)** | | |
| --- | --- | --- |
| S13 | S3 AND S11 AND S12 | 18 |
| S12 | S7 OR S10 | 7,265 |
| S11 | mechanical OR biomechanical OR thickness OR depth OR diameter OR stiffness OR hardness OR elasticity OR strain | 19,434 |
| S10 | S8 OR S9 | 1,406 |
| S9 | calcaneal tendon | 1 |
| S8 | Achilles | 1,405 |
| S7 | S4 OR S5 OR S6 | 5,924 |
| S6 | skin OR epidermis OR dermis OR cutaneous OR subcutaneous | 5,916 |
| S5 | pedal soft tissues | 0 |
| S4 | plantar soft tissues | 8 |
| S3 | S1 OR S2 | 837 |
| S2 | diab* feet | 23 |
| S1 | diab* foot | 829 |

| 1. **ProQuest (Allied and Nursing)** 2. **ProQuest (Health and Medical)** | | |
| --- | --- | --- |
| S6 | 1 AND 4 AND 5 | 107 |
| S5 | 2 OR 3 | 218,222 |
| S4 | noft(mechanical OR biomechanical OR thickness OR depth OR diameter OR stiffness OR hardness OR elasticity OR strain) | 458,654 |
| S3 | noft(Achilles OR “calcaneal tendon”) | 4,325 |
| S2 | noft(“plantar soft tissues” OR “pedal soft tissues” OR skin OR epidermis OR dermis OR cutaneous OR subcutaneous) | 241,205 |
| S1 | noft(“diab* foot” OR “diab* feet”) | 3,066 |

| 1. **Web of Science** | | |
| --- | --- | --- |
| 6 | #5 AND #4 AND #1 | 403 |
| 5 | #3 OR #2 | 843,720 |
| 4 | TOPIC: (mechanical OR biomechanical OR thickness OR depth OR diameter OR stiffness OR hardness OR elasticity OR strain) | 4,410,404 |
| 3 | TOPIC: (Achilles OR “calcaneal tendon”) | 14,215 |
| 2 | TOPIC: (“plantar soft tissues” OR “pedal soft tissues” OR skin OR epidermis OR dermis OR cutaneous OR subcutaneous) | 830,263 |
| 1 | TOPIC: (“diab* foot” OR “diab* feet”) | 10,135 |
