# Supplementary material for: Plantar soft tissues and Achilles tendon thickness and stiffness in people with diabetes: a systematic review
Source: J Foot Ankle Res. 2021 Apr 28;14:35. doi: 10.1186/s13047-021-00475-7 (PMC8080343; doi:10.1186/s13047-021-00475-7)
Supplement: Supplementary file 2 — Additional file 2. Reasons for article exclusion. [file 13047_2021_475_MOESM2_ESM.docx]

**Additional file 2: Reasons for article exclusion**

| **No.** | **Author (year)** | **Title** | **Reason for exclusion** |
| --- | --- | --- | --- |
| 1 | Teoh (2020) | Identification of potential plantar ulceration among diabetes patients using plantar soft tissue stiffness | Single cohort |
| 2 | Seixas (2019) | Relationship between skin temperature and soft tissue hardness in diabetic patients: an exploratory study | Single cohort |
| 3 | Cuaderes (2009) | Weight-bearing activity and foot parameters in Native Americans with diabetes with and without foot sensation | Single cohort |
| 4 | Payne (2002) | Determinants of plantar pressures in the diabetic foot | Single cohort |
| 5 | Abouaesha (2001) | Plantar tissue thickness is related to peak plantar pressure in the high-risk diabetic foot | Single cohort |
| 6 | Seirafi (2009) | Biophysical characteristics of skin in diabetes: a controlled study | Wrong anatomical location |
| 7 | Yoon (2002) | Quantitative measurement of desquamation and skin elasticity in diabetic patients | Wrong anatomical location |
| 8 | de Jonge (2015) | Achilles tendons in people with type 2 diabetes show mildly compromised structure: an ultrasound tissue characterisation study | Wrong outcomes |
| 9 | Abate (2013) | Ultrasound morphology of the Achilles in asymptomatic patients with and without diabetes | Wrong outcomes |
| 10 | Chao (2011) | A novel noncontact method to assess the biomechanical properties of wound tissue | Wrong outcomes |
| 11 | Tajaddini (2007) | Laser-induced auto-fluorescence (LIAF) as a method for assessing skin stiffness preceding diabetic ulcer formation | Wrong outcomes |
| 12 | Charanya (2004) | Effect of foot sole hardness, thickness and footwear on foot pressure distribution parameters in diabetic neuropathy | Wrong outcomes |
| 13 | Gefen (2003) | Plantar soft tissue loading under the medial metatarsals in the standing diabetic foot | Wrong outcomes |
| 14 | van Schie (2002) | The effect of silicone injections in the diabetic foot on peak plantar pressure and plantar tissue thickness: a 2-year follow-up | Wrong outcomes |
| 15 | Hsu (2002) | ﻿Biomechanics of the heel pad for type 2 diabetic patients | Wrong outcomes |
| 16 | Ito (2017) | Dynamic measurement of surface strain distribution on the foot during walking | Wrong patient population |
| 17 | Trindade (2014) | Development of a Wearable Ultrasonic Sensor and Method for Continuous Monitoring of Mechanical Properties of Plantar Soft Tissue for Diabetic Patients | Wrong patient population |
| 18 | Yarnitzky (2006) | Real-time subject-specific monitoring of internal deformations and stresses in the soft tissues of the foot: A new approach in gait analysis | Wrong patient population |
| 19 | Gefen (2001) | In vivo biomechanical behaviour of the human heel pad during the stance phase of gait | Wrong patient population |
| 20 | Wang (1999) | Ultrasonographic measurement of the mechanical properties of the sole under the metatarsal heads | Wrong patient population |
| 21 | Williams (2017) | A preliminary study of patient-specific mechanical properties of diabetic and healthy plantar soft tissue from gated magnetic resonance imaging | Wrong study design |
| 22 | Zabihollahy (2016) | Continuous Monitoring of Mechanical Properties of Plantar Soft Tissue for Diabetic Patients using Wearable Ultrasonic and Force Sensors | Wrong study design |
| 23 | Tahrani (2012) | Cutaneous Structural and Biochemical Correlates of Foot Complications in High-Risk Diabetes | Wrong study design |
| 24 | Yudovsky (2011) | Monitoring temporal development and healing of diabetic foot ulceration using hyperspectral imaging | Wrong study design |
| 25 | Cuaderes (2009) | Reliability and limitations of the durometer and PressureStat to measure plantar foot characteristics in Native Americans with diabetes | Wrong study design |
| 26 | Singh (2007) | Multiprobe laser reflectometry in imaging and characterization of biological tissues | Wrong study design |
| 27 | Scarton (2006) | Towards the generation of a parametric foot model using principal component analysis: A pilot study | Wrong study design |
| 28 | Gefen (2001) | Integration of plantar soft tissue stiffness measurements in routine MRI of the diabetic foot | Wrong study design |
| 29 | Zheng (1999) | Indentation assessment of plantar foot tissue in diabetic patients | Wrong study design |
| 30 | Grant (1997) | Electron microscopic investigation of the effects of diabetes mellitus on the Achilles tendon | Wrong study design |
| 31 | Buschmann (1995) | Histology and histomorphometric Analysis of the Normal and Atrophic Heel Fat Pad | Wrong study design |
| 32 | Jahss (1992) | Investigations into the Fat Pads of the Sole of the Foot: Heel Pressure Studies | Wrong study design |
| 33 | Bennett (1990) | The mechanical properties of the human subcalcaneal fat pad in compression | Wrong study design |
