# Supplementary material for: Plantar soft tissues and Achilles tendon thickness and stiffness in people with diabetes: a systematic review
Source: J Foot Ankle Res. 2021 Apr 28;14:35. doi: 10.1186/s13047-021-00475-7 (PMC8080343; doi:10.1186/s13047-021-00475-7)
Supplement: Supplementary file 3 — Additional file 3. Plantar tissue thickness values. [file 13047_2021_475_MOESM3_ESM.docx]

**Additional file 3: Plantar tissue thickness values (Mean ± SD, mm)**

| **No.** | **Author** | **Method of measurement** | **Distance between skin and bone** | **Hallux** | **MTH1** | **MTH2** | **MTH3** | **MTH4** | **MTH5** | **Heel** |
| --- | --- | --- | --- | --- | --- | --- | --- | --- | --- | --- |
| 1 | Lechner (2019) | Epidermal-dermal junction zone | X | — | Ctrls: 0.88±0.26  T2DM: 0.78±0.24 | — | — | — | — | Ctrls: 0.93±0.30  T2DM: 0.92±0.23 |
| 2 | Oh (2018) | Vertical distance between skin and MTH | ✓ | — | Ctrls: 8.69±3.88  T2DM: 6.4±2.07 | Ctrls: 10.64±3.66  T2DM: 8.85±3.65 | Ctrls: 9.21±3.07  T2DM: 8.15±3.44 | Ctrls: 8.54±2.67  T2DM: 7.38±3.2 | Ctrls: 6.74±2.59  T2DM: 5.72±3.51 | — |
| 3 | Kumar (2015) | Not defined in words - but shown in a figure.  Skin = Epidermis and dermis; plantar fat pad, plantar fascia and intrinsic foot muscles measured separately. | X | — | Skin  Ctrls: 0.24±0.05  T2DM: 0.17±0.03  Fat pad  Ctrls: 0.49±0.12  T2DM: 0.39±0.11  (Discrete values not available for T2-DPN group) | Skin  Ctrls: 0.26±0.04  T2DM: 0.19±0.05  Fat pad  Ctrls: 0.65±0.19  T2DM: 0.53±0.16  (Discrete values not available for T2-DPN group) | Skin  Ctrls: 0.26±0.04  T2DM: 0.19±0.05  Fat pad  Ctrls: 0.63±0.16  T2DM: 0.52±0.14  (Discrete values not available for T2-DPN group) | Skin  Ctrls: 0.26±0.04  T2DM: 0.19±0.06  Fat pad  Ctrls: 0.59±0.15  T2DM: 0.47±0.14  (Discrete values not available for T2-DPN group) | Skin  Ctrls: 0.25±0.46  T2DM: 0.19±0.07  Fat pad  Ctrls: 0.54±0.13  T2DM: 0  42±0.11  (Discrete values not available for T2-DPN group) | — |
| 4 | Chatzistergos (2014) | Bone-to-probe distance | Distance between probe and bone | — | — | — | — | — | — | Ctrls: 19.5±4.7  T2DM: 19.4±3.5 |
| 5 | Cheing (2013) | Determined from the time of the flight of the US signal reflecting from the soft tissue-bone interface and associated with the speed of sound in soft tissues. | ✓ | (Discrete values not available; data presented in a bar chart) | (Discrete values not available; data presented in a bar chart) | — | (Discrete values not available; data presented in a bar chart) | — | (Discrete values not available; data presented in a bar chart) | (Discrete values not available; data presented in a bar chart) |
| 6 | Jan (2013) | Distance between the first and second echo, i.e. between the transducer-skin and soft tissue-bone interface. | ✓ | — | Ctrls: 13.6±1.4  T2DM: 13.5±2.3 | — | — | — | — | — |
| 7 | Chao (2012) | Determined from the distance between the demarcation echo lines:   - Epidermis (broad echo-rich band) and - upper dermis (thin echolucent band) | X | Epidermis  Ctrls: 0.50±0.16  T2DM: 0.54±0.18  DFU: 0.40±0.13  Upper dermis:  Ctrls: 0.17±0.08  T2DM: 0.19±0.07  DFU: 0.28±0.09 | — | — | — | — | — | — |
| 8 | Chao (2011) | For US (epidermis only):  Determined from the distance between the demarcation echo lines: between the soft tissue-bone interface  For TUPS (total PTT):  The time-of-flight of the ultrasound echo signal required to propagate from the plantar skin surface to that soft tissue-bone interface was used to calculate the tissue thickness. A uniform sound speed of 1540 m/s in soft tissue was assumed. | X  For US  ✓  For TUPS | Epidermis  Ctrls: 0.51±0.17  T2DM: 0.55±0.18  T2-DPN: 0.45±0.11  DFU: 0.43±0.14  Total PTT  Ctrls: 7.11±1.63  T2DM: 7.711.58  T2-DPN: 8.04±1.37  DFU: 8.64±1.15 | Epidermis  Ctrls: 0.55±0.19  T2DM: 0.56±0.18  T2-DPN: 0.51±0.17  DFU: 0.51±0.16  Total PTT  Ctrls: 9.43±1.88  T2DM: 9.95±1.63  T2-DPN: 10.21±2.67  DFU: 10.07±2.26 | — | Epidermis  Ctrls: 0.62±0.15  T2DM: 0.66±0.15  T2-DPN: 0.56±0.18  DFU: 0.51±0.17  Total PTT  Ctrls: 9.58±1.55  T2DM: 10.51±2.00  T2-DPN: 10.92±1.62  DFU: 10.92±1.52 | — | Epidermis  Ctrls: 0.61±0.13  T2DM: 0.68±0.13  T2-DPN: 0.58±0.17  DFU: 0.52±0.19  Total PTT  Ctrls: 8.38±2.06  T2DM: 8.87±2.08  T2-DPN: 10.02±1.68  DFU: 9.53±2.35 | Epidermis  Ctrls: 0.66±0.13  T2DM: 0.68±0.15  T2-DPN: 0.58±0.14  DFU: 0.53±0.15  Total PTT  Ctrls: 23.65±3.29  T2DM: 25.73±2.54  T2-DPN: 25.02±3.19  DFU: 25.12±3.10 |
| 9 | Sun (2011) | The total thickness of the plantar soft tissues between the bony surface and the skin surface of the measurement site was determined from the flight time of the ultrasound echo signal that reflected from soft tissue–bone interface. | ✓ | Ctrls: 4.76±0.87  T2-DPN: 5.16±0.18 | Ctrls: 7.67±0.35  T2-DPN: 8.25±0.28 | Ctrls: 8.74±0.36  T2-DPN: 8.88±0.28 | — | — | — | Ctrls: 18.87±0.65  T2-DPN: 18.25±0.52 |
| 10 | Hsu (2009) | Distance between the skin and the cortex of the calcaneus | ✓ | — | — | — | — | — | — | Ctrls: 1.84±1.2  T2DM: 1.93±0.30 |
| 11 | Hsu (2007) | Measured from the skin surface to the nearest metatarsal head cortex on the sonogram | ✓ | — | Ctrls: 1.41  (SEM 0.05)  T2DM: 1.37  (SEM 0.06) | Ctrls: 1.35 (SEM 0.07)  T2DM: 1.34 (SEM 0.09) | Ctrls: 1.26 (SEM 0.04)  T2DM: 1.28 (SEM 0.09) | Ctrls: 1.16 (SEM 0.03)  T2DM: 1.27 (SEM 0.10) | Ctrls: 1.12  (SEM 0.07)  T2DM: 1.27 (SEM 0.06) | — |
| 12 | Hashmi (2006) | Determined from the change in echogenicity; epidermis only | X | — | — | — | Ctrls: 0.185  T2-DPN: 0.20  (Discrete values for T2DM without DPN group not available) | — | — | — |
| 13 | Mueller (2003) | Distance from the most distal aspect of the metatarsal head to the inner surface of the skin; lateral view. | ✓ | — | Ctrls: 14.5±2.6  DM: 13.7±3.3 | Ctrls: 9.9±2.3  DM: 11.0±2.9 | Ctrls: 8.8±1.9  DM: 8.5±2.1 | Ctrls: 8.0±2.0  DM: 7.9±1.7 | Ctrls: 7.2±1.9  DM: 7.4±1.5 | — |
| 14 | Thomas (2003) | Points of max convexity of met heads and/or calcaneum located. The measurement of gel/skin interface to the bone was noted. | ✓ | Ctrls: 6.4±1.0  T2DPN: 5.9±0.5  DFU: 10.4±2.9 | — | Ctrls: 10.7±1.4  T2-DPN: 9.7±1.3  DFU: 14.9 | Ctrls: 8.5±1.8  T2-DPN: 8.3±1.4  DFU: 10.2 | | | Ctrls: 14.6±3.1  T2-DPN: 14.3±1.5  DFU: 22.6 |
| 15 | Robertson (2002) | Distance from the most plantar bone at the head (or sesamoid) to the inner surface of the skin; lateral view. | ✓ | — | Ctrls: 10±2  DM: 10±1 | | | | | — |
| 16 | Hsu (2000) | Distance between the skin and the calcaneus | ✓ | — | — | — | — | — | — | Ctrls: 1.65±0.19  T2DM: 1.72±0.31  DFU: 1.78±0.37 |
| 17 | Zheng (2000) | Determined from the flight time of the US echo signal that reflected from soft tissue-bone interface. The sound speed in soft tissues was assumed uniformly to be 1540 m/s. | ✓ | Young ctrls: 9±1  Elderly DPN: 7±2 | Young ctrls: 12±2  Elderly DPN: 7±2 | Young ctrls: 14±0.5  Elderly DPN: 8±2 | — | — | — | Young ctrls: 16±2  Elderly DPN: 12±3 |
| 18 | Brink (1995) | Distance between the skin surface and bone echo | ✓ | — | Young ctrls: 9.5 (SEM 0.5)  Old ctrls: 6.1  (SEM 0.7)  DM: 5.9  (SEM 0.6) | Young ctrls: 8.1  (SEM 0.3)  Old ctrls: 6.6  (SEM 0.5)  DM: 7.6 (SEM 0.4) | Young ctrls: 7.5 (SEM 0.3)  Old ctrls: 7.7 (SEM 0.3)  DM: 6.8  (SEM 0.3) | Young ctrls: 6.9  (SEM 0.3)  Old ctrls: 7.0 (SEM 0.2)  DM: 6.7 (SEM 0.4) | Young ctrls: 6.0 (SEM 0.2)  Old ctrls: 5.5 (SEM 0.3)  DM: 5.6 (SEM 0.3) | Young ctrls: 15.4 (SEM 0.5)  Old ctrls: 15.4 (SEM 0.7)  DM: 13.7  (SEM 0.8) |
| 19 | Gooding (1986) | From the skin surface to the soft tissue-bone interface; from transverse scans. | ✓ | — | Ctrls: 12.92  (SEM 0.42)  DM: 11.60  (SEM 0.29)  DFU: 10.67  (SEM 0.56) | Ctrls: 14.17 (SEM 0.26)  DM: 12.70 (SEM 0.29)  DFU: 12.48 (SEM 0.72) | Ctrls: 13.56 (SEM 0.29)  DM: 13.06 (SEM 0.24)  DFU: 12.34 (SEM 0.54) | Ctrls: 12.91 (SEM 0.34)  DM: 12.10 (SEM 0.26)  DFU: 11.05 (SEM 0.53) | Ctrls: 11.47 (SEM 0.27)  DM: 10.70 (SEM 0.23)  DFU: 10.72 (SEM 0.38) | Ctrls: 18.62 (SEM 0.36)  DM: 17.33  (SEM 0.29)  DFU: 15.77 (SEM 0.39) |
| 20 | Gooding (1985) | Calcaneal-soft tissue junction located; measurements taken from transverse scans of the mid-heel. | Not specified | — | — | — | — | — | — | Ctrls: 16.6  (SEM 0.32)  DM: 17.8  (SEM 0.31) |
| **Symbols:** ✓: Yes; X: No; —: Not examined.  **Abbreviations:** Ctrl, Group of non-diabetic controls; DFU, Group with diabetic foot ulcer; DM, Group with diabetes mellitus; DPN, Group with diabetic peripheral neuropathy; MTH, Metatarsal head; PTT, Plantar tissue thickness; SD, Standard deviation; SEM, Standard error of mean; T2, Type 2 diabetes only; TUPS, Tissue ultrasound palpation system; US, Ultrasonography. | | | | | | | | | | |
