# Supplementary material for: Plantar soft tissues and Achilles tendon thickness and stiffness in people with diabetes: a systematic review
Source: J Foot Ankle Res. 2021 Apr 28;14:35. doi: 10.1186/s13047-021-00475-7 (PMC8080343; doi:10.1186/s13047-021-00475-7)
Supplement: Supplementary file 4 — Additional file 4. Achilles tendon thickness values. [file 13047_2021_475_MOESM4_ESM.docx]

**Additional file 4: Achilles tendon thickness values (mean ± SD, mm)**

| **No.** | **Author (year)** | **Method of measurement** | **Proximal** | **Mid-portion** | **Distal** |
| --- | --- | --- | --- | --- | --- |
| 1 | Harish (2020) | AP diameter | Ctrls: 2.9±0.6  T2DM: 3.3±0.7  T2-DPN: 3.1±0.8 | Ctrls: 4.4±0.6  T2DM: 4.7±0.8  T2-DPN: 4.8±0.7 | Ctrls: 3.9±0.6  T2DM: 4.6±1.0  T2-DPN: 4.5±1.0 |
| 2 | İyidir (2019) | AP diameter | – | Ctrls: 4.6±0.5 (R); 4.6±0.1 (L)  T2DM: 5.0±0.5 (R); 5.0±0.1 (L)  T2-DPN: 5.0±0.6 (R); 5.2±0.1 (L) | – |
| 3 | Evranos (2015) | AP diameter | Ctrls: 1.8±0.3 (R); 1.80±0.2 (L)  T2DM: 1.8±0.3 (R); 1.9±0.4 (L)  T2DFU: 1.9±0.3 (R); 2.0±0.8 (L) | Ctrls: 4.2± 0.6 (R); 4.5±0.7 (L)  T2DM: 4.5±0.7 (R); 4.7±0.6 (L)  T2DFU: 5.2±0.8 (R); 5.2±0.6 (L) | Ctrls: 4.1±0.6 (R); 4.2±0.6 (L)  T2DM: 4.5±0.8 (R); 4.6±0.7 (L)  T2DFU: 5.4± 1.9 (R); 5.2±0.7 (L) |
| 4 | Cheing (2013) | From the superior surface of the tendon to its insertion at the posterior calcaneus | – | – | Ctrls: 6.1±1.3  T2DM: 6.9±1.0  T2-DPN: 8.3±1.3 |
| 5 | Papanas (2009) | Max AP diameter | Ctrls: 7.2±1.8  T2DM: 7.2±0.8  T2-DPN: 7.1±1.2 | | |
| 6 | Batista (2008) | – | Ctrls: 5.9 (range: 4.0 to 8.2)  DM: 5.0±0.8 (range: 3.8 to 7.6) | | |
| 7 | Akturk (2007) | Max AP diameter | – | Ctrl female: 4.3±0.5 (R); 4.2±0.4 (L)  Ctrl male: 5.1±1.0 (R); 5.1±0.8 (L)  T2DM female: 5.1±0.7 (R); 5.3±0.8 (L)  T2DM male: 5.1±0.7 (R); 5.2±0.5 (L) | – |
| 8 | D’ambrogi (2005) | – | – | – | Ctrls: 4.0±0.5  DM: 4.6±1.0  DPN: 4.9±1.7  DFU: 5.2±1.7 |
| 9 | Giacomozzi (2005) | – | – | – | Ctrls: 4.0±0.5  DM: 4.6±1.0  DPN: 4.9±1.7  DFU: 5.2±1.7 |
| **Symbols:** —: Not examined / not stated.  **Abbreviations:** AP, Anteroposterior; Ctrls, Group of non-diabetic controls; DFU, Group with diabetic foot ulcerations; DM, Group with diabetes mellitus; DPN, Group with diabetic peripheral neuropathy; L, Left; R, Right; SD, Standard deviation; T2, Participants with Type 2 diabetes only. | | | | | |
