# Supplementary material for: Plantar soft tissues and Achilles tendon thickness and stiffness in people with diabetes: a systematic review
Source: J Foot Ankle Res. 2021 Apr 28;14:35. doi: 10.1186/s13047-021-00475-7 (PMC8080343; doi:10.1186/s13047-021-00475-7)
Supplement: Supplementary file 5 — Additional file 5. Directionality of findings for plantar soft tissues. [file 13047_2021_475_MOESM5_ESM.docx]

# Additional file 5: Directionality of findings for plantar soft tissues

|  |  | |  | **Anatomical regions examined** | | | | | | | | | | | | | **Comparison groups** | | | | | | | | |  | | |
| --- | --- | --- | --- | --- | --- | --- | --- | --- | --- | --- | --- | --- | --- | --- | --- | --- | --- | --- | --- | --- | --- | --- | --- | --- | --- | --- | --- | --- |
| **No.** | **Author** | | **Assessment tool** | **Hallux** | | **MTH1** | | **MTH2** | **MTH3** | | **MTH4** | | **MTH5** | | **Heel** | | **Ctrls** | | **DM** | | **DPN** | | | **DFU** | | **Findings** | | |
| **Plantar tissue thickness** | | | | | | | | | | | | | | | | | | | | | | | | | | | |  |
| 1 | Lechner (2019) | | OCT | — | ↑ NS (p=0.56) | | — | | | — | | — | — | ↑ NS (p=0.31) | | ✓ | | ✓ T2 | | | | | — | | — | | ↑ NS  Heel > MTH1 |  |
| 2 | Oh (2018) | | MRI | — | ↓ (p=0.001) | | ↓ (p=0.017) | | | ↓ NS (p=0.111) | | ↓ (p=0.047) | ↓ NS (p=0.105) | — | | ✓ | | ✓  T2 | | | | | — | | — | | ↓ Except at MTH5 |  |
| 3 | Kumar (2015) | | US | — | ↓ Skin  (p<0.001)  ↓ Plantar fat pad (p=0.002)  T2-DPN ≈ T2DM | | ↓ Skin (p<0.001)  ↓ Plantar fat pad (p=0.008)  T2-DPN ≈ T2DM | | | ↓ Skin (p<0.001)  ↓ Plantar fat pad (p=0.005)  T2-DPN ≈ T2DM | | ↓ Skin (p<0.001)  ↓ Plantar fat pad (p<0.001)  T2-DPN ≈ T2DM | ↓ Skin (p<0.001)  ↓ Plantar fat pad (p<0.001)  T2-DPN ≈ T2DM | — | | ✓ | | ✓ T2 | | | | | ✓ T2 | | — | | ↓  T2-DPN ≈ T2DM  ↓ also observed in plantar fascia thickness and intrinsic muscles bulk |  |
| 4 | Chatzistergos (2014) | | US probe connected with a dynamometer | — | — | | — | | | — | | — | — | NS (p=0.985) | | ✓ | | ✓ T2 | | | | | | | — | | NS |  |
| 5 | Cheing (2013) | | TUPS | ↑ NS (p-values not stated) | ↑ NS (p-values not stated) | | — | | | ↑ NS (p-values not stated) | | — | ↑ NS (p-values not stated) | ↑ NS (p-values not stated) | | ✓ | | ✓ T2 | | | | | ✓ T2 | | — | | ↑ NS (p-values not stated);  T2-DPN > T2DM > ctrls (all NS) |  |
| 6 | Jan (2013) | | TUPS | — | NS (p=0.943) | | — | | | — | | — | — | — | | ✓ | | — | | | | | ✓ T2 | | — | | NS |  |
| 7 | Chao (2012) | | US | Epidermis: T2DM > DFU and ctrls (p=0.009);  Upper dermis:  DFU > T2DM and ctrls (p<0.001) | — | | — | | | — | | — | — | — | | ✓ | | ✓ T2 | | | | | ✓ T2 Active or history of DFU | | | | Anatomical and group variation |  |
| 8 | Chao (2011) | | US  (epidermis only);  TUPS (total PTT) | Epidermis: Overall trend: T2DM > ctrls > T2-DPN > DFU;  T2DM > DPN and DFU  (p = 0.022)  Total PTT: Overall trend: ↑ all T2DM groups;  Ctrls < DFU (p=0.004) | Epidermis: NS (p=0.650)  Total PTT: NS (p=0.463) | | — | | | Epidermis: Overall trend: T2DM > ctrls > T2-DPN > DFU;  DFU < T2DM (p=0.012)  Total PTT: Overall trend: ↑ all T2DM groups;  Ctrls < DPN and DFU (p=0.006) | | — | Epidermis: Overall trend: T2DM > ctrls > T2-DPN > DFU;  DFU < T2DM (p=0.04)  Total PTT: Overall trend: ↑ all T2DM groups;  Ctrls < DPN (p=0.014) | Epidermis: Overall trend: T2DM > ctrls > T2-DPN > DFU;  DFU < T2DM and ctrls  (p=0.001)  Total PTT: Overall trend: ↑ all T2DM groups;  ctrls < T2DM (p=0.020) | | ✓ | | ✓ T2 | | | | | ✓ T2 | | ✓ T2 Active or history of DFU | | Epidermis: T2DM > ctrls > T2-DPN > DFU; Except MTH1  Total PTT: ↑ T2DM, T2-DPN and DFU; Except MTH1 |  |
| 9 | Sun (2011) | | TUPS | NS (p=0.067) | NS (p=0.257) | | NS (p=0.696) | | | — | | — | — | NS (p=0.252) | | ✓ | | — | | | | | ✓ T2 | | — | | NS |  |
| 10 | Hsu (2009) | | US-based loading device | — | — | | — | | | — | | — | — | NS (p=0.222) | | ✓ | | ✓ T2 | | | | | | | — | | NS  Microchamber ≈ macrochamber |  |
| 11 | Hsu (2007) | | US with a load cell | — | NS (p=0.778) | | NS (p=0.438) | | | NS (p=0.351) | | NS (p=0.035) | NS (p=0.270) | — | | ✓ | | ✓ T2 | | | | | | | — | | NS  MTH1 > MTH2 > MTH3 > MTH4 > MTH5 (all NS) |  |
| 12 | Hashmi (2006) | | US | — | — | | — | | | T2-DPN > ctrls (p=0.017)  Ctrls > T2DM (p=0.024) | | — | — | — | | ✓ | | ✓ T2 | | | | | | | — | | T2-DPN > ctrls > T2DM |  |
| 13 | Mueller (2003) | | SXCT | — | NS (p-values not stated) | | NS (p-values not stated) | | | NS (p-values not stated) | | NS (p-values not stated) | NS (p-values not stated) | — | | ✓ | | — | | ✓ History of DFU | | | | | | | NS |  |
| 14 | Thomas (2003) | | US | Active DFU > (T2-DPN ≈ ctrls) (p<0.005) | — | | NS (p-values not stated) | | | NS (p-values not stated) | | | | NS (p-values not stated) | | ✓ | | — | | ✓ T2 | | | | | ✓ T2 Active DFU | | Anatomical variation  DFU sites > non-ulcerated sites |  |
| 15 | Robertson (2002) | | CT | — | NS (p=0.62) | | | | | | | | | — | | ✓ | | — | | ✓ History of DFU | | | | | | | NS |  |
| 16 | Hsu (2000) | | US | — | — | | — | | | — | | — | — | ↑ NS (p=0.254) | | ✓ | | ✓ T2 | | | | | | | ✓ T2 With active forefoot DFU | | ↑ NS  DFU > T2DM > ctrls (all NS) |  |
| 17 | Zheng (2000) | | US indentation system | ↓  (p<0.05) | ↓  (p<0.05) | | ↓  (p<0.05) | | | — | | — | — | ↓  (p<0.05) | | ✓Young | | — | | | | ✓Elderly | | | — | | ↓ |  |
| 18 | Brink (1995) | | US | — | NS (p-values not stated) | | NS (p-values not stated) | | | NS (p-values not stated) | | NS (p-values not stated) | NS (p-values not stated) | NS (p-values not stated) | | ✓ | |  | | | | ✓ History of DFU | | | | | NS  Previous DFU sites < non-ulcerated sites |  |
| 19 | Gooding (1986) | | US | — | ↓ DM (p<0.025)  ↓ DFU  (p<0.05) | | ↓ DM (p<0.025)  ↓ DFU  (p<0.05) | | | NS (p-values not stated) | | NS (p-values not stated) | NS (p-values not stated) | ↓ DM (p<0.01)  ↓ DFUs (p<0.01) | | ✓ | | ✓ | | | | | | ✓ Active or history of DFU | | | Anatomical variation |  |
| 20 | Gooding (1985) | | US | — | — | | — | | | — | | — | — | ↑ (p<0.01) | | ✓ | | ✓ | | | | | | — | | | ↑ |  |
| **Plantar tissue stiffness** | | | | | | | | | | | | | | | | | | | | | | | | | | | |  |
| 1 | Kwak (2020) | Subject-specific finite element model | | — | — | | | — | | — | | — | — | | Skin: ↓  T2DM < younger ctrls (p<0.05); (T2DM not compared with older ctrls)  Fat: ↑ T2DM > Older ctrls and younger ctrls (p<0.05) | ✓Young and older | | ✓ T2 | | | | | | | — | | Skin: ↓ Fat: ↑ |  |
| 2 | Lechner (2019) | Cutometer | | — | ↑ (p=0.001) | | | — | | — | | — | — | | NS (p=0.21) | ✓ | | ✓ T2 | | | | — | | | — | | Anatomical variation  Heel > MTH |  |
| 3 | Chatzistergos (2014) | US probe connected with a dynamometer | | — | — | | | — | | — | | — | — | | ↑ Stiffness  (p=0.022 or p=0.034)†  ↑ Normalised stiffness (p=0.038 or p=0.001)† | ✓ | | ✓ T2 | | | | | | | — | | ↑ |  |
| 4 | Cheing (2013) | TUPS | | ↑ T2DM (p=0.02)  ↑ T2-DPN (p<0.05) | ↑  T2DM (p=0.03)  NS for  T2-DPN (p=0.38) | | | — | | ↑  T2DM (p=0.03)  ↑  T2-DPN (p<0.05) | | — | ↑  T2DM (p=0.02)  ↑  T2-DPN (p<0.05) | | NS for T2DM (p=0.40)  ↑ T2-DPN (p<0.05) | ✓ | | ✓ T2 | | | | ✓ T2 | | | — | | Anatomical and group variation |  |
| 5 | Jan (2013) | TUPS | | — | ↑ (p=0.004) | | | — | | — | | — | — | | — | ✓ | | — | | | | ✓ T2 | | | — | | ↑ |  |
| 6 | Periyasamy (2012) | Durometer | | ↑  T2DM  (Right: p<0.005; Left: p<0.05)  ↑  T2-DPN (Right & Left: p<0.05) | ↑  T2DM  (Right: p<0.001; Left: p<0.05)  ↑  T2-DPN (Right & Left: p<0.05) | | | NS for DM and DPN  (p values not stated) | | ↑  T2DM (Right & Left: p<0.01)  ↑  T2-DPN (Right & Left: p<0.05) | | | | | ↑  T2DM  (Right & Left, medial & lateral: NS)  ↑  T2-DPN (Right medial: p<0.05; Right lateral: p<0.01; Left medial: p<0.01; Left lateral: p<0.05) | ✓ | | ✓ T2 | | | | ✓ T2 | | | — | | ↑ Except MTH2 |  |
| 7 | Chao (2011) | TUPS | | ↑ All three T2DM groups;  Ctrls < DFU (p=0.012) | ↑ All three T2DM groups  Ctrls < DFU (p=0.029) | | | — | | ↑ All three T2DM groups  Ctrls < DFU (p=0.010) | | — | ↑ All three T2DM groups  Ctrls < DM and DFU (p=0.002) | | ↑ All three T2DM groups  Ctrls < DPN and DFU (p=0.003) | ✓ | | ✓ T2 | | | | ✓ T2 | | | ✓ T2 Active or history of DFU | | ↑ |  |
| 8 | Sun (2011) | TUPS | | ↑ (p<0.001) | ↑ (p=0.009) | | | ↑ (p<0.001) | | — | | — | — | | ↑ (p=0.003) | ✓ | | — | | | | ✓ T2 | | | — | | ↑ |  |
| 9 | Hsu (2009) | US with load cell | | — | — | | | — | | — | | — | — | | NS for heel pad; (p=0.191)  Microchamber: T2DM > ctrls (p=0.001); Macrochamber: Ctrls > T2DM (p<0.001) | ✓ | | ✓ T2 | | | | | | | — | | NS  Anatomical variation |  |
| 10 | Cheung (2006) | MRE | | — | — | | | — | | — | | — | — | | ↑ NS (p-values not stated) | ✓ | | — | | | | ✓ | | | — | | ↑ NS |  |
| 11 | Hashmi (2006) | Cutometer | | — | — | | | — | | ↑ Series elastic element on retraction (p=0.04)  ↓ Epidermal plasticity (p = 0.007) | | — | — | | — | ✓ | | ✓ T2 | | | | | | | — | | Variation with outcome metric |  |
| 12 | Puri (2005) | Durometer | | A channel:  NS  H channel: ↑ (p<0.01) | A channel: ↑ (p<0.005)  H channel: ↑ (p<0.0001) | | | — | | A channel: ↑ (p < 0.0003)  H channel: ↑ (p<0.0001) | | | | | A channel: ↑ Medial heel (p<0.0003); Lateral heel (p<0.0003)  H channel: ↑ Medial heel (p<0.0001); Lateral heel (p<0.0002) | ✓ | | ✓ | | | | | | | — | | Variation with outcome metric |  |
| 13 | Mueller (2003) | SXCT | | — | ↑ (Nil inferential statistics) | | | — | | ↑ (Nil inferential statistics) | | — | ↑ (Nil inferential statistics) | | — | ✓ | | — | | | | ✓ History of DFU | | | | | ↑ (Nil inferential statistics) |  |
| 14 | Thomas (2003) | Durometer | | ↑  Active DFU (p<0.002)  NS for  T2-DPN (p-values not stated) | — | | | ↑ NS  T2-DPN and DFU (p-values not stated) | | ↑ NS  T2-DPN and DFU (p-values not stated) | | | | | ↑ NS  T2-DPN and DFU (p-values not stated) | ✓ | | — | | | | ✓ T2 | | | ✓ T2 Active DFU | | ↑ NS Except for Hallux in active DFU group;  DFU sites > non-ulcerated sites |  |
| 15 | Klaesner (2002) | Indentor system | | — | ↑ K1 only (p<0.05)  NS for K2 (p-values not stated) | | | — | | ↑ K1 only (p<0.05)  NS for K2 (p-values not stated) | | — | ↑  K1 and K2 (p<0.05) | | NS  K1 and K2 (p-values not stated) | ✓ | | — | | | | ✓ History of DFU | | | | | Variation with outcome metric |  |
| 16 | Hsu (2000) | US | | — | — | | | — | | — | | — | — | | Elastic Modulus: NS  (p=0.647)  Compressibility Index: NS (p=0.657) | ✓ | | ✓ T2 | | | | | | | ✓ T2 Forefoot DFU | | NS |  |
| 17 | Zheng (2000) | US indentation system | | ↑ (p values not stated) | ↑ (p values not stated) | | | ↑ (p values not stated) | | — | | — | — | | ↑† (p values not stated) | ✓ Young | | — | | | | ✓ Elderly | | | — | | ↑  For elderly DPN: MTH1 > (Hallux ≈ MTH2 ≈ Heel)  For young controls:  NS between all tested sites |  |
| 18 | Piaggesi (1999) | Durometer | | — | — | | | — | | — | | — | — | | For medial and lateral heel  DPN > (DM ≈ Ctrls) (p<0.01) | ✓ | | ✓ | | | | ✓ | | | — | | DPN > (DM ≈ Ctrls) |  |
| 19 | Brink (1995) | Durometer | | — | ↑ NS (p-values not stated) | | | ↑ (p<0.05) | | ↑ (p<0.05) | | ↑ (p<0.05) | ↑ NS (p-values not stated) | | ↓ NS (p-values not stated) | ✓ | | — | | | | ✓ History of recurrent DFU | | | | | Anatomical variation  MTHs > Heel;  DFU sites > non-ulcerated sites |  |
| **Symbols:** †: Discrepancy identified in-text; ↑: Significantly increased; ↓: Significantly decreased; ✓: Data available; –: Not examined; ≈: No significant differences; >: Greater than; <: Less than.  **Abbreviations**: CT, Computed tomography; Ctrls, Group of non-diabetic controls; DFU, Group with diabetic foot ulcer; DM, Group with diabetes mellitus; DPN, Group with diabetic peripheral neuropathy; MRE, Magnetic resonance elastography; MRI, Magnetic resonance imaging; MTH, Metatarsal head (plantar); NS, No significant differences; OCT, Optical coherence tomography; PTT, Plantar tissue thickness; SXCT, Spiral X-ray computed tomography; T2, Type 2 diabetes only; TUPS, Tissue ultrasound palpation system; US, Ultrasonography. | | | | | | | | | | | | | | | | | | | | | | | | | | | |  |
