# Supplementary material for: Plantar soft tissues and Achilles tendon thickness and stiffness in people with diabetes: a systematic review
Source: J Foot Ankle Res. 2021 Apr 28;14:35. doi: 10.1186/s13047-021-00475-7 (PMC8080343; doi:10.1186/s13047-021-00475-7)
Supplement: Supplementary file 6 — Additional file 6. Directionality of findings for Achilles tendon. [file 13047_2021_475_MOESM6_ESM.docx]

# Additional file 6: Directionality of findings for Achilles tendon

|  |  |  | **Anatomical regions examined** | | | **Comparison groups** | | | |  |
| --- | --- | --- | --- | --- | --- | --- | --- | --- | --- | --- |
| **No.** | **Author** | **Assessment tool** | **Proximal** | **Mid-portion** | **Distal** | **Ctrl** | **DM** | **DPN** | **DFU** | **Findings** |
| **Achilles tendon thickness** | | | | | | | | | | |
| 1 | Harish (2020) | US | ↑ T2DM and  T2-DPN  (p<0.001)  T2DM ≈ T2-DPN (p>0.05) | ↑ T2DM and  T2-DPN (p<0.001)  T2DM ≈ T2-DPN (p>0.05) | ↑ T2DM and  T2-DPN (p<0.001)  T2DM ≈ T2-DPN (p>0.05) | ✓ | ✓ T2  (7/81 with DFUs; 8 with leg amputations) | ✓ T2  (with symptoms suggestive of; DPN not objectively tested) | — | ↑ |
| 2 | İyidir (2019) | US | — | ↑ T2DM  (p=0.006;  Right: p=0.02; Left: p<0.001)  ↑ T2-DPN  (p=0.01;  Right: p=0.02; Left: p<0.001)  T2DM ≈ T2-DPN (p=0.991) | — | ✓ | ✓ T2 | ✓ T2 | — | ↑ |
| 3 | Evranos (2015) | US | T2-DFU > (T2DM ≈ ctrls) (p=0.029) | T2-DFU > (T2DM ≈ ctrls) (p<0.001) | T2-DFU > (T2DM ≈ ctrls) (p<0.001) | ✓ | ✓ T2 | | ✓ T2 Active DFU  (23% with bilateral DFUs; 43% with osteomyelitis) | T2-DFU > (T2DM ≈ ctrls) |
| 4 | Cheing (2013) | TUPS | — | — | ↑  T2DM  (p<0.05)  ↑  T2DPN > T2DM (p<0.001) | ✓ | ✓ T2 | ✓ T2 | — | ↑ T2-DPN > T2DM > ctrls |
| 5 | Papanas (2009) | MRI | NS for T2DM and T2-DPN vs ctrls (p=0.194);  NS for T2DM vs ctrl men (p=0.935); ↑ for T2DM vs ctrl women (p=0.030);  Severe T2-DPN ≈ moderate T2-DPN (p=0.870) | | | ✓ | ✓ T2 | ✓ T2 | — | T2DM ≈ ctrls;  Sex variation;  Severe DPN ≈ moderate DPN |
| 6 | Batista (2008) | US | ↓ (Nil inferential statistics) | | | ✓ | ✓ | | — | ↓ (Nil inferential statistics) |
| 7 | Akturk (2007) | US | — | NS for T2DM vs ctrl men (p=0.74);  ↑ for T2DM vs ctrl women (p<0.001);  NS for T2DM women vs men (p=0.73);  NS for T2-DPN women vs men  (p>0.05)  ↓ for ctrl women vs men (p=0.002)  ↑ for T2-DPN vs T2DM men (p=0.019);  NS for T2-DPN vs T2DM women  (p-values not stated) | — | ✓ | ✓ T2 | | — | Sex variation |
| 8 | D’ambrogi (2005) | US | — | — | ↑  DM (p=0.016);  DPN (p=0.026); DFU (p=0.002);  DPN ≈ DM (p-values not stated) | ✓ | ✓ | ✓ | ✓ History of DFU (up to 3 months) | ↑ All DM groups;  DPN ≈ DM;  ↑ plantar fascia thickness observed |
| 9 | Giacomozzi (2005) | US | — | — | ↑  DPN (p<0.05); DFU (p<0.05);  NS for DM (p-values not stated) | ✓ | ✓ | ✓ | ✓ History of DFU (up to 3 months) | ↑ for DPN and DFU; NS for DM;  ↑ plantar fascia thickness observed |
| **Achilles tendon stiffness** | | | | | | | | | | |
| 1 | Harish (2020) | SWEI | — | — | ↓ (p<0.001)  T2DPN ≈ T2DM (p>0.05) | ✓ | ✓ T2  (7/81 with DFUs; 8 with leg amputations) | ✓ T2  (with symptoms suggestive of; DPN not objectively tested) | — | ↓ |
| 2 | İyidir (2019) | ARFI | — | NS T2DM ≈ ctrls (p=0.993)  ↓ T2-DPN (p<0.001) | — | ✓ | ✓ T2 | ✓ T2 | — | (T2DM ≈ ctrls) > T2-DPN |
| 3 | Couppé (2016) | US | — | — | ↑ Young’s Modulus at common force (p<0.01);  NS Young's modulus at max force; (p-values not stated)  NS for absolute stiffness (p-values not stated) | ✓ | ✓ | | — | Variation with outcome metric |
| 4 | Evranos (2015) | SE | NS  (p=0.37) | (T2DM ≈ controls) > DFU (p<0.001) | (T2DM ≈ controls) > DFU (p=0.027) | ✓ | ✓ T2 | | ✓ T2  (23% with bilateral DFUs; 43% with osteomyelitis) | Anatomical variation |
| 5 | Cheing (2013) | TUPS | — | — | ↑ NS for DM and DPN (p=0.71) | ✓ | ✓ T2 | ✓ T2 | — | ↑ NS |
| **Symbols:** ↑: Significantly increased; ↓: Significantly decreased; ✓: Data available; –: Not examined; ≈: No significant differences; >: Greater than; <: Less than.  **Abbreviations**: ARFI, Acoustic Radiation Force Impulse; Ctrls, Group of non-diabetic controls; DFU, Group with diabetic foot ulcerations; DM, Group with diabetes mellitus; DPN, Group with diabetic peripheral neuropathy; MRI, Magnetic resonance imaging; NS, No significant differences; SE, Strain elastography; SWEI, Shear wave elasticity imaging; T2, Type 2 diabetes only; TUPS, Tissue ultrasound palpation system; US, Ultrasonography. | | | | | | | | | | |
